# A randomised controlled trial to compare clinical and cost-effectiveness of an online parent-led treatment for child anxiety problems with usual care in the context of COVID-19 delivered in Child and Adolescent Mental Health Services in the UK (Co-CAT): a study protocol for a randomised controlled trial

**DOI:** 10.1186/s13063-022-06833-5

**Published:** 2022-11-16

**Authors:** Lucy Taylor, Sophie Giles, Sophie Howitt, Zoe Ryan, Emma Brooks, Lucy Radley, Abigail Thomson, Emily Whitaker, Fauzia Knight, Claire Hill, Mara Violato, Polly Waite, Vanessa Raymont, Ly-Mee Yu, Victoria Harris, Nicola Williams, Cathy Creswell

**Affiliations:** 1 Departments of Experimental Psychology and Psychiatry, University of Oxford, Oxford, UK; 2 School of Psychology and Clinical Language Sciences, University of Reading, Reading, UK; 3 Health Economics Research Centre, Nuffield Department of Population Health, NIHR Oxford Biomedical Research Centre, University of Oxford, Oxford, UK; 4 Department of Psychiatry, University of Oxford, Oxford, UK; 5 Oxford Primary Care Clinical Trials Unit, Nuffield Department of Primary Care Health Sciences, University of Oxford, Oxford, UK

**Keywords:** COVID-19, Digital health, Randomised controlled trial, Child anxiety, CAMHS, Parent-led, Intervention, CBT

## Abstract

**Background:**

In the context of COVID-19, NHS Child and Adolescent Mental Health Services (CAMHS) and other children’s mental health services have faced major challenges in providing psychological treatments that (i) work when delivered remotely and (ii) can be delivered efficiently to manage increases in referrals as social distancing measures have been relaxed. Anxiety problems are a common reason for referral to CAMHS, children with pre-existing anxiety problems are particularly vulnerable in the context of COVID-19, and there were concerns about increases in childhood anxiety as schools reopened. The proposed research will evaluate the clinical and cost-effectiveness of a brief online parent-led cognitive behavioural treatment (CBT) delivered by the OSI (Online Support and Intervention for child anxiety) platform with remote support from a CAMHS therapist compared to ‘COVID-19 treatment as usual’ (C-TAU) in CAMHS and other children’s mental health services throughout the COVID-19 pandemic.

**Methods:**

We will conduct a two-arm, multi-site, randomised controlled non-inferiority trial to evaluate the clinical and cost-effectiveness of OSI with therapist support compared to CAMHS and other child mental health services ‘COVID-19 treatment as usual’ (C-TAU) during the COVID-19 outbreak and to explore parent and therapists’ experiences.

**Discussion:**

If non-inferiority is shown, the research will provide (1) a solution for efficient psychological treatment for child anxiety disorders while social distancing (for the COVID-19 context and future pandemics); (2) an efficient means of treatment delivery as ‘normal service’ resumes to enable CAMHS to cope with the anticipated increase in referrals; and (3) a demonstration of rapid, high-quality evaluation and application of online interventions within NHS CAMHS to drive forward much-needed further digital innovation and evaluation in CAMHS settings. The primary beneficiaries will be children with anxiety disorders and their families, NHS CAMHS teams, and commissioners who will access a potentially effective, cost-effective, and efficient treatment for child anxiety problems.

**Trial registration:**

ISRCTN ISRCTN12890382. Registered prospectively on 23 October 2020.

**Supplementary Information:**

The online version contains supplementary material available at 10.1186/s13063-022-06833-5.

## Administrative information

Note: the numbers in curly brackets in this protocol refer to SPIRIT checklist item numbers. The order of the items has been modified to group similar items (see http://www.equator-network.org/reporting-guidelines/spirit-2013-statement-defining-standard-protocol-items-for-clinical-trials/).

**Table:** 

Title {1}	A randomised controlled trial to compare clinical and cost-effectiveness of an online parent-led treatment for child anxiety problems with usual care in the context of COVID-19 delivered in Child and Adolescent Mental Health Services in the UK (Co-CAT): A study protocol for a randomised controlled trial
Trial registration {2a and 2b}.	ISRCTN12890382 (registered prospectively 23/10/2020) 10.1186/ISRCTN12890382
Protocol version {3}	V2.3, 15.07.2022
Funding {4}	DHSC/UKRI COVID-19 Rapid Response Initiative NIHR Policy Response Programme (NIHR204435- PRP (ST-01-42) CAMHS) Oxford and Thames Valley NIHR Applied Research Collaboration Oxford Health NIHR Biomedical Research Centre
Author details {5a}	Lucy Taylor *Departments of Experimental Psychology and Psychiatry, University of Oxford Email: lucy.taylor@psych.ox.ac.uk Sophie Giles Departments of Experimental Psychology and Psychiatry, University of Oxford Email: sophie.giles@psych.ox.ac.uk Sophie Howitt Departments of Experimental Psychology and Psychiatry, University of Oxford Email: sophie.howitt@psych.ox.ac.uk Zoe Ryan Departments of Experimental Psychology and Psychiatry, University of Oxford Email: zoe.ryan.psych.ox.ac.uk Emma Brooks Departments of Experimental Psychology and Psychiatry, University of Oxford Email: emma.brooks@psych.ox.ac.uk Lucy Radley Departments of Experimental Psychology and Psychiatry, University of Oxford Email: lucy.radley@psych.ox.ac.uk Abigail Thomson Departments of Experimental Psychology and Psychiatry, University of Oxford Email: abigail.thomson@psych.ox.ac.uk Emily Whitaker Departments of Experimental Psychology and Psychiatry, University of Oxford Email: emily.whitaker@psych.ox.ac.uk Dr Fauzia Knight Departments of Experimental Psychology and Psychiatry, University of Oxford Email: fauzia.knight@psych.ox.ac.uk Dr Claire Hill School of Psychology and Clinical Language Sciences, University of Reading Email: claire.hill@reading.ac.uk Dr Mara Violato Health Economics Research Centre, Nuffield Department of Population Health, University of Oxford Email: mara.violato@dph.ox.ac.uk Dr Polly Waite Departments of Experimental Psychology and Psychiatry, University of Oxford Email: polly.waite@psych.ox.ac.uk Dr Vanessa Raymont Department of Psychiatry, University of Oxford Email: vanessa.raymont@psych.ox.ac.uk Dr Ly-Mee Yu Oxford Primary Care Clinical Trials Unit, Nuffield Department of Primary Care Health Sciences, University of Oxford Email: ly-mee.yu@phc.ox.ac.uk Victoria Harris Oxford Primary Care Clinical Trials Unit, Nuffield Department of Primary Care Health Sciences, University of Oxford Email: victoria.harris@phc.ox.ac.uk Nicola Williams Oxford Primary Care Clinical Trials Unit, Nuffield Department of Primary Care Health Sciences, University of Oxford Email: nicola.williams@phc.ox.ac.uk Professor Cathy Creswell Departments of Experimental Psychology and Psychiatry, University of Oxford Email: cathy.creswell@psych.ox.ac.uk *Corresponding author (lucy.taylor@psych.ox.ac.uk)
Name and contact information for the trial sponsor {5b}	University of Oxford Joint Research Office 1^st^ floor, Boundary Brook House Churchill Drive Headington Oxford, OX3 7GB E-mail: ctrg@admin.ox.ac.uk
Role of sponsor {5c}	The funder and sponsor have no role in the trial design, collection, management, analysis, and interpretation of data, or reporting of the study. The funder and sponsor have no role in the decision to submit the report for publication.

## Introduction

### Background and rationale {6a}

#### Background

More than a quarter of the population will experience an anxiety disorder at some point during their life, and for half of these people, this first occurs by the age of 11 years [1]. Anxiety disorders in childhood often continue into adolescence and adulthood and put these children at increased risk for other serious mental health disorders and impaired quality of life in adulthood [2]. As a result, societal costs for anxiety disorders are substantial, with estimated total costs in England of £8.9 billion, expected to rise to £14.2 billion by 2026 [3].

Cognitive behavioural therapy (CBT) for children with anxiety disorders has been shown to be effective [4], but only a minority of children with anxiety disorders access treatment [5, 6]. A recent UK survey found that more than 60% of children with anxiety disorders had not received any professional support, and only 2% had received CBT [7]. Families face extensive barriers accessing professional support for child anxiety disorders, including high demands on services, limited available support, and long waiting lists [8, 9].

Traditional CBT for child anxiety disorders is typically lengthy (e.g. 10–20 h of therapist contact [4]) and involves specialists working directly with the child. We have developed a briefer version of the traditional treatment that involves working directly with parents/carers (hereafter ‘parents’), and supporting them to help their child overcome their difficulties with anxiety. This brief parent-guided treatment has similar outcomes to the traditional approach despite being delivered with about 5 h of therapist contact and can be delivered by non-specialists [10, 11]. However, improving treatment efficiency further could enable more families to access effective treatment when they first need it. Online delivery of parent-guided treatment offers a means to do this by substantially reducing the amount of therapist contact time needed. Delivering treatment online also has the potential to increase access to families who may experience barriers to accessing traditional treatment approaches. In a recent survey of parents of children with elevated anxiety in England, all parents had some form of Internet access, and more than 85% of parents reported that online treatment delivery would reduce stigma for families and allow families to use it at any time, and from home [12].

We have worked in collaboration with families, NHS clinicians, and a tech-company to co-design an online version of our parent-guided treatment for child anxiety disorders called OSI (Online Support and Intervention for child anxiety) [13]. OSI comprises a parent website, an accompanying therapist case management system, and an accompanying child game app (see https://osiresearch.org.uk/osi/). Modules are supported by 7 × 20-min telephone sessions between the parent and a therapist and a review session 4 weeks after the final treatment session.

#### Importance in the context of COVID-19

The Health Innovation Network [14] highlighted that children with existing anxiety issues are a high-risk population during the COVID-19 pandemic, and the UK Co-SPACE study, which has been tracking child and adolescent mental health throughout the pandemic, identified high levels of fear and worry about COVID-19 among children, including fears about leaving the house, and a significant increase in emotional symptoms in primary school-aged children during lockdown [15]. Child and adolescent mental health services (CAMHS) and parents raised concerns about an increase in child anxiety at key stress points during the pandemic, including as restrictions eased [14].

In the Co-SPACE study, parents reported that they wanted help via online materials and personalised support from a professional [16]; however, there are limited evidence-based platforms available to CAMHS to do this [17]. During the pandemic CAMHS therapists moved to typically delivering ‘face to face’ therapy via phone/videocall but have had little evidence-based guidance about how to do this most effectively and efficiently [18]. OSI provides a potential means to address the challenges of meeting the needs of children with anxiety problems and their families; it can be delivered as intended despite social distancing measures and is sufficiently flexible to address COVID-19-specific fears/worries. However, it has not yet been subject to systematic evaluation, and we do not know whether outcomes are as good as those CAMHS are currently achieving through their usual practice and whether OSI with therapist support enables further efficiencies.

### Objectives {7}

The proposed research will evaluate the clinical and cost-effectiveness of OSI with therapist support for the treatment of child anxiety problems, compared to ‘COVID-19 treatment as usual’ (C-TAU) in CAMHS and associated children’s mental health services (from here on ‘CAMHS’) throughout the next phases of the COVID-19 pandemic. Further aims are to explore the trajectory of change as reported within the OSI platform, to inform further developments, and to understand therapist and parents’ experiences of treating child anxiety problems (across both arms) in the current context. This will maximise learning to (a) enable rapid implementation of remote treatment delivery in CAMHS in any subsequent periods of social distancing and (b) maintain the use of online platforms (such as OSI) in CAMHS as ‘normal service’ resumes.

### Trial design {8}

We will conduct a two-arm, multi-site, randomised controlled non-inferiority trial to evaluate the clinical and cost-effectiveness of OSI with therapist support compared to CAMHS ‘COVID-19 treatment as usual’ (C-TAU) during the COVID-19 pandemic and to explore parent and therapists’ experiences. The study procedure is in line with the Standard Protocol Items: Recommendations for Interventional Trials (SPIRIT) statement 2013 [19].

## Methods: participants, interventions, and outcomes

### Study setting {9}

The study will take place in child and adolescent mental health services (CAMHS) across the NHS and Local Authorities in the UK, including Third Sector organisations that provide child mental health care on behalf of the NHS/Local Authorities. A list of study sites is available on the study website [20].

We will invite NHS Child and Adolescent Mental Health Services, Local Authority, and Third Sector organisations that provide child mental health care on behalf of the NHS/Local Authorities to be trial sites in the study. We will invite teams to join the study through the following routes: (i) direct contact to teams that have expressed an interest in the study/OSI or have participated in previous randomised controlled trials, (ii) contact through the NHS Future Collaboration Platform (Parent Led CBT workspace), (iii) contact through local and regional networks and collaborations (AHSN, children and young people’s IAPT training centres), and (iv) NIHR clinical research network support teams. Teams will be provided with a study flyer to give a brief overview of the study. Where services include School Mental Health Support Teams, there is an additional school flyer that can be provided to schools to help identify eligible participants.

### Eligibility criteria {10}

Study participants are children aged 5–12 years where anxiety is the primary presenting problem (as determined by clinical teams), and their parents.

#### Inclusion criteria

- *Child:* (1) be aged 5–12 years at intake, (2) have a primary problem of anxiety, and (3) be willing and able to assent.
- *Parent*: (1) have sufficient English language to complete measures/access interventions, (2) have access to the Internet, and (3) be willing and able to provide consent.
- *Therapists*: (1) provide psychological treatment to children in participating services and (2) be willing and able to provide informed consent.

#### Exclusion criteria

Participants are not eligible if ANY of the following apply:

- *Child*: (1) has co-morbid conditions that are likely to interfere with treatment delivery (established autism spectrum condition/learning disability, suicidal intent/recurrent or potentially life-limiting self-harm), (2) is identified by social services due to child protection concerns, or (3) is identified via a Schools Team and is in Reception, year 1 or year 2 in a school that is taking part in the MY-CATS (ISRCTN Registration Number: 82398107) study (another study where the child may receive the OSI intervention).
- *Parent*: (1) has a significant intellectual impairment or severe mental health problem that is likely to interfere with treatment delivery or (2) is unable to access or understand the written English language materials necessary for the interventions.
- *Therapists*: There are no exclusion criteria for therapists.

### Who will take informed consent? {26a}

Parents will provide online consent for themselves and their child, and children will provide online assent via a secure online system.

Although the child may choose not to provide any data directly (if they do not wish to complete any of the child report measures), both parent consent and child assent are required for randomisation. We anticipate that some participating children will need adult support to understand the study information and assent and to complete the measures and we will provide parents with guidance on how to do this with instructions available on the online system.

Participant information (parent/child/therapist) will be provided in written and video form (for parents and children) providing information on the exact nature of the study; what it will involve for the participant; the implications and constraints of the protocol; and the known side effects and any risks involved in taking part. It will be clearly stated that the participant is free to withdraw from the study at any time for any reason without prejudice to future care, without affecting their legal rights, and with no obligation to give the reason for withdrawal. Each potential participant will be given sufficient time to read through the participant information sheet and ask questions, either to the clinical contact or the research team, before deciding whether to take part.

If clinical teams have not been notified that a participating family has consented/assented to the study within 2 weeks of approach, they will be free to contact the participant to arrange alternative treatment. Clinical teams will be notified 2 weeks before the overall study recruitment window is coming to an end so that they can inform any families that have not yet responded how much time they have left to consent/assent should they wish to participate.

Participants must provide their name and date of the latest approved version of the informed consent form (via a unique link) before any study-specific procedures are performed. A copy of the signed informed consent/assent forms will be available to be downloaded at each timepoint when the parent accesses the system and notice of this will also be emailed to the local investigator (or their delegated authority), who will also have access to download these from the system to add them to the child’s medical record.

Therapists invited to take part in a qualitative interview will be provided with a study information sheet by email and/or via the study website. A privacy notice will also be supplied. Consent will be obtained from the therapist at the time of interview, prior to audio-recording commencing. The researcher will read the consent clauses and record their responses in writing. They will email a copy of the consent form securely to the participating therapist.

### Additional consent provisions for collection and use of participant data and biological specimens {26b}

This trial does not involve collecting biological specimens.

### Interventions

#### Explanation for the choice of comparators {6b}

The comparator is COVID-19 treatment as usual, i.e. whatever treatment the participating services are delivering to treat child anxiety problems in the COVID-19 context.

#### Intervention description {11a}

The study intervention is OSI (Online Support and Intervention for child anxiety) with therapist support. OSI is an online adaptation of an evidence-based brief parent-guided CBT programme for the treatment of anxiety problems in preadolescent children. OSI comprises a parent website, an accompanying therapist case management system, and an accompanying child game app (see https://osiresearch.org.uk/osi/). The 7 modules are supported by weekly* 20-min telephone/video call sessions between the parent and a therapist and a review session 4 weeks after the final treatment session (*although this may be adjusted by parents/clinicians if required).

#### Criteria for discontinuing or modifying allocated interventions {11b}

During the course of the study, a participant may choose to withdraw early from the study treatment at any time. This may happen for several reasons, including but not limited to:

- The occurrence of what the participant perceives as an intolerable adverse event (AE)
- Inability to comply with the study procedures
- Participant decision

In the case of participant withdrawal, we will retain data collected only until that point unless the participant requests otherwise or agrees to take part in further assessments.

It is also possible that the clinical team might withdraw the participant from the research treatment (OSI+therapist support) if allocated. If the therapist delivering treatment feels the child should not continue with OSI+therapist support (e.g. due to serious comorbidities arising that need to be addressed), we will retain the data and continue to invite the participant to take part in further assessments, unless the participant requests otherwise.

The number of withdrawals from treatment and/or follow-up measures will be logged with a summary of their reasons (if offered by the participant).

#### Strategies to improve adherence to interventions {11c}

Therapists will receive a video-based training programme (45 min) and a treatment manual prior to delivering treatment within the study. All teams will be offered regular question and answer sessions throughout the treatment delivery phase to support set-up and delivery. Clinical supervision will be provided within teams following their usual procedures.

#### Relevant concomitant care permitted or prohibited during the trial {11d}

There are no exclusion criteria relating to concomitant care, but families are not invited to take part if they are part of the MY-CATS study to avoid receiving the intervention twice (see above).

#### Provisions for post-trial care {30}

Participants remain under the clinical care of the service that they are being treated in, which will arrange further treatment or discharge from the service according to their local practices.

### Outcomes {12}

#### Primary measures and objective

*Primary objective:* To evaluate the parent-reported clinical effectiveness of a brief parent-led cognitive behavioural treatment (CBT) delivered by the OSI platform with therapist support (OSI+therapist support) for the treatment of child anxiety compared to COVID-19 treatment as usual (C-TAU) in CAMHS throughout the next phases of the COVID-19 pandemic.

*Primary outcome measure*: The Child Anxiety Impact Scale-parent report (CAIS-P) captures the degree to which anxiety is interfering in the child and family’s life, measured at 26 weeks post-randomisation.

#### Secondary measures and objectives

*Secondary clinical outcomes*: Child-reported anxiety interference (CAIS-C) and anxiety symptoms (RCADS-C). Parent-reported child anxiety symptoms (RCADS-P, SCAS-8P), overall functioning (ORS), COVID-19-specific worries, and common comorbid emotional and behavioural problems (SDQ-P). All will be measured at 14 and 26 weeks post-randomisation.

*Secondary clinical objective:* Further assessment of the clinical effectiveness of OSI+therapist support for the treatment of child anxiety compared to COVID-19 treatment as usual (C-TAU) in CAMHS throughout the next phases of the COVID-19 pandemic.

*Economic outcomes*: Parent quality of life (EQ-5D-5L, parent-self report); child quality of life (CHU-9D proxy version, i.e. parent-report on child); and the Child Anxiety Impact Scale- parent report (CAIS-P). School attendance (parent-reported actual school attendance as a percentage of expected school attendance). Therapist logs of time spent on treatment delivery. Measured at 14 and 26 weeks post-randomisation.

*Economic objective*: To evaluate the cost-effectiveness of OSI+therapist support for the treatment of child anxiety compared to COVID-19 treatment as usual (C-TAU) in CAMHS.

#### Exploratory measures and objectives

*Exploratory measures:* The following measures are built in to OSI to assess parent-reported child anxiety symptoms (RCADS-P; SCAS-8P), interference (CAIS-P), global (ORS) and goal-based (GBOs) outcomes, and parent experience of the intervention (SRS), during weeks 1–7 of OSI treatment (modules 0–6) to explore the trajectory of change reported within the OSI arm.

*Exploratory objective*: To monitor child outcomes on a session-by-session basis within the novel treatment arm.

*Treatment experience:* We will conduct qualitative interviews with parents and therapists/supervisors between 14 and 26 weeks post-randomisation, and also use a therapist ‘experience of treatment’ questionnaire, measured at the end of treatment.

*Objectives:* To understand therapist and parents’ experiences of treating child anxiety problems in the current context.

*Use of the online intervention:* OSI incorporates routine outcome measures that are used for clinical purposes, but are also available to the research team through the OSI researcher portal in pseudo-anonymised form. Usage data are also available through the OSI researcher portal that provides detailed information on parents’ use of the website: frequency of sessions on the website, time spent on the website, and also time spent on the different activities.

*Objectives:* This information will be used to describe the use of and compliance with the intervention.

#### Adverse event measures

Clinicians are requested to monitor and report any harms and adverse events during the treatment phase. Additionally, parents and children are given the opportunity to report any negative impact of participating in the study as part of the 14- and 26-week post-randomisation assessments, using a self-report form.

See below for more detail on the measures.

### Participant timeline {13}

Where parents and their children provide online consent or assent, respectively, they will be asked to complete online baseline assessments (all questionnaires), prior to randomisation. After randomisation, parents will be asked to complete a further short questionnaire about treatment expectations and acceptability. Treatment in both arms will be organised by the clinical teams who will be requested to start as soon as possible and at most within 12 weeks of randomisation. Families allocated to the intervention arm will access OSI and a member of the clinical team will provide support as they work through each OSI module. Participating parents and children in both arms (OSI+therapist support and C-TAU) will be sent a link to complete post-treatment and follow-up assessments (questionnaires) online 14 and 26 weeks after randomisation.

Therapists will provide information on COVID-19 treatment as usual for each participant at the start of their treatment within the therapist treatment logs in order for us to be able to describe what services are delivered in the COVID-19 context, specifically the treatment approach being followed, who they are having contact with (e.g. child, parent, both), and for how long.

Therapists will provide brief demographic information at the study outset, will record information on activity with each participating family, and will complete a brief questionnaire about their experience of the treatment after completing treatment with each participating family.

We will also conduct qualitative interviews with a subgroup of parents and therapists/supervisors (*n*= 25–40), between 14 and 26 weeks after randomisation, purposively sampled on the basis of demographics, and we will continue to sample to ensure variability in treatment outcomes across both arms, if possible, to explore and to understand therapist and parents’ experiences of treating child anxiety in this context.

SPIRIT schedule of enrolment, interventions, and assessments

**Table:** 

		Enrolment			Post-allocation		Close-out
			After consent	After randomisation	Treatment	14 weeks after randomisation	26 weeks after randomisation
Timepoint:			Baseline			Post-treatment	Follow-up
Enrolment	Eligibility screen	X					
	Informed consent	X					
	Allocation			X			
	Demographic information		X				
Interventions	OSI				X		
	C-TAU				X		
Assessments							
Child report							
Symptom measure	RCADS-C		X			X	X
Functional impairment	CAIS-C		X			X	X
Adverse Events	AE Self-report					X	X
Parent report							
Symptom measures	RCADS-P		X			X	X
	SCAS-P-8		X			X	X
Functional impairment	CAIS-P		X			X	X
	ORS		X			X	X
Co-morbid problems	SDQ-P		X			X	X
Pandemic Anxiety Scale	PAS		X			X	X
Treatment acceptability	Credibility and Expectation of Improvement scale			X		X	
Adverse events	AE Self-report					X	X
Health economics	CSRI		X			X	X
	EQ-5D-5L- P		X			X	X
	CHU-9D (YP proxy)		X			X	X
	CAIS-P		X			X	X
OSI+therapist support ARM ONLY Measures collected during treatment (parent only)	RCADS-P				X		
	SCAS-P-8				X		
	CAIS-P				X		
	ORS				X		
	SRS				X		
	GBOs				X		
Qualitative interviews						X (subgroup of participants interviewed once each between 14 and 26 weeks).	
Therapist Logs					X	X	X

### Sample size {14}

Between 418 and 560 children (209–280 per group) with a primary anxiety problem and their parents will be randomised across the two treatment arms. This sample size is considered to be sufficient to provide a standardised noninferiority margin = 0.33 and between 80 and 90% power (allowing for 30% attrition). The required sample size was calculated using PASS 2019.

A sub-sample of up to 20–40 parents and therapists/supervisors from both arms will be involved in qualitative interviews to explore their experiences of treatment. We will purposively sample from parents who have reached 14 weeks post-randomisation until we reach saturation in terms of (i) representation across the sample for geographic, economic, and educational backgrounds and (ii) until no new themes are identified. We will also aim to recruit therapists who reflect differing characteristics (e.g. service provider type and location, demographic factors, and clinical backgrounds), as well as therapists’ supervisors. We have previously found that this number of participants is typically sufficient for data saturation.

### Recruitment {15}

Participating families will be identified by their clinical teams. Clinical teams will follow their usual procedures to identify the child’s primary presenting problem. The clinical team will conduct an initial assessment of eligibility according to the inclusion and exclusion criteria. Where the clinical team identifies that the study inclusion criteria are met (and exclusion criteria are not), they will briefly outline the study to the parents. If parents express an interest in the study, they will be asked to provide an email address to which they will be sent a secured link to the study IT system where the parent will be able to access online information sheets which will provide further information about the study and what would be expected of the participants, alongside contact details for the study team (email and phone number) so that they can be contacted to answer any questions. There will also be video information (information sheet text being read aloud verbatim). In addition, there is dedicated, age-appropriate, online information including a video (consisting of the information sheet with text being read aloud verbatim) for the child. The parent will then be invited to provide online consent for themselves and their child. Where parents provide consent, a further email will be sent with a link for the child to provide assent.

Therapists whose role includes delivery of psychological interventions to children with anxiety problems within participating clinical teams will be invited to take part and consenting families will be allocated to these therapists for treatment. Allocation of families to therapists will be managed within participating clinical teams.

## Assignment of interventions: allocation

### Sequence generation {16a}

Participants will be randomised in a 1:1 ratio to (i) OSI+therapist support or (ii) CAMHS treatment as usual for child anxiety problems within the COVID-19 context (C-TAU).

Minimisation by child age (≤8; ≥9), gender, service type (school based or not school based), and baseline anxiety-associated interference including permuted block size will be used to ensure balance across groups.

### Concealment mechanism {16b}

Participants will be randomised using a fully validated and secured web-based randomisation system called Sortition that will automatically occur after the participating parent completes the consent and baseline measures, and the child completes assent (online).

### Implementation {16c}

Sortition will automatically send an email, including the result of the allocation treatment arm, to the trial team, the clinical team, and the participants.

## Assignment of interventions: blinding

### Who will be blinded {17a}

Due to the nature of the trial, blinding to condition is not possible to the trial participants or research team; however, the statistical analyses will be conducted blind to trial condition.

### Procedure for unblinding if needed {17b}

There are no planned interim analyses so unblinding of statisticians is not anticipated prior to the datalock.

## Data collection and management

### Plans for assessment and collection of outcomes {18a}

Assessments will be completed via online self-report questionnaires, administered at baseline, 14 weeks post-randomisation (post-treatment) and 26 weeks post-randomisation (follow-up). The research team will keep careful track of assessment completion and will contact participants (by email, text, phone) if measures have not been completed within 3 days of the invitation to encourage completion of measures (at all timepoints).

A summary of the measures that are provided at each timepoint is given in the ‘Participant timeline {13} section.

#### Demographic information

Participating child and parent demographics will be collected based on parent report. The data collected will include (i) parent age and child date of birth, (ii) parent and child gender, (iii) parent and child ethnicity, (iv) child use of medication, (v) parental education, (vi) parental employment status and occupation, (vii) household circumstances, (viii) employment and income details, and (ix) child’s school type and education provision. This information will be used to describe the sample, inform randomisation minimisation, and inform the health economic evaluation.

In order to describe therapists who delivered the treatment in this study, therapists will provide information on (i) their age, (ii) gender, (iii) ethnicity, (iv) professional background, (v) years qualified and of clinical experience, (vi) current working arrangements, (vii) experience of working with children with anxiety problems, (viii) relevant training, and (ix) preferred ways of working with children with anxiety problems and their families.

#### Impact of child anxiety

The Child Anxiety Impact Scale- parent report (CAIS-P/C): The CAIS-P/C will be used to determine the extent to which anxiety interferes with the child’s life. This measure covers three psychosocial domains (academic, social activities, and home/family environments) and consists of 27 items rated on a 4-point scale. An additional 4 ‘global’ items assess overall interference. There are versions for children and parents to complete, both of which have been shown to have good psychometric properties [21, 22]. The CAIS-P/C will be completed at baseline and then at 14 and 26 weeks post-randomisation by both parent and child.

#### Symptoms of child anxiety

Revised Child Anxiety and Depression Scale-Child and Parent version (RCADS-C/P): The RCADS-C/P is routinely used within CAMHS. It is a 47-item questionnaire, with corresponding child-report and parent-report versions that assess symptoms of separation anxiety disorder, social anxiety disorder, generalised anxiety disorder, panic disorder, obsessive compulsive disorder, and major depressive disorder. Responders rate how often each item applies on a 0 (‘never’) to 3 (‘always’) scale. The RCADS-C/P has been shown to have robust psychometric properties in children from age 7 [23, 24]. RCADS-C/P will be completed at baseline, and then at 14 and 26 weeks post-randomisation by both parent and child.

Brief Spence Children’s Anxiety Scale-Parent Version (SCAS-P-8): The SCAS-P-8 is a brief version of the Spence Children’s Anxiety Scale [25]. It is an 8-item questionnaire designed to assess symptoms of anxiety disorders in children. An initial evaluation of the questionnaire indicates it has good psychometric properties in children from ages 7 to 11 [25]. Only 1 of the 8 items is required to be collected to score this measure as 7/8 items overlap with those already collected within the RCADS-P. The additional item that enables us to calculate a SCAS-P-8 total score will be completed at baseline and then at 14 and 26 weeks post-randomisation by the parent and is added as an additional item at the end of the RCADS-P questionnaire.

#### Overall functioning

Outcome Rating Scale (ORS): The ORS [26] will be used to assess functioning across different areas of the child’s life. It comprises four simple rating scales in which the parent rates how their child has been feeling over the last week (individually, interpersonally, socially, and overall wellbeing). Each item is rated using a 10-cm visual analogue scale, with instructions to place a mark on each line. A higher score indicates better functioning. It has good reliability and validity [27]. The ORS will be completed at baseline and then at 14 and 26 weeks-post randomisation by the parent.

#### Common comorbid emotional and behavioural problems

Strengths and Difficulties Questionnaire-parent report (SDQ-P): The SDQ-P [28] is a behavioural screening questionnaire. It comprises 5 scales assessing emotional symptoms, conduct problems, hyperactivity/inattention, peer relationship problems, and prosocial behaviour. It has satisfactory reliability [29, 30] and good concurrent and discriminant validity [31, 32]. The parent-report version will be completed at baseline and then at 14 and 26 weeks-post randomisation.

#### COVID-19-specific worries

Pandemic Anxiety Scale (PAS): The PAS [33] is a parent report 7-item scale designed to capture specific aspects of the COVID-19 pandemic that are provoking anxiety in the child. It comprises two factors: disease anxiety (e.g. catching, transmitting the virus) and consequence anxiety (e.g. impact on economic prospects). An initial evaluation of the scale indicates that the PAS is a reliable and valid parent report measure about their child [33]. The PAS will be completed by the parent at baseline and then at 14 and 26 weeks post-randomisation.

#### Health economic measures

EQ-5D (5L) (parent self-report): The EQ-5D (5L) will be used to assess parent’s quality of life [34]. It is a well-validated measure of health-related quality of life, designed to estimate quality-adjusted life years (QALYs), that is widely used across disease areas. It contains five questions, each related to a different domain of everyday life (mobility, self-care, usual activities, pain/discomfort, anxiety/depression). For each domain, the respondent has to indicate whether they experience no problems, slight problems, moderate problems, severe problems, or extreme problems. It also includes a visual analogue scale (VAS) for participants to rate their overall health on a scale from 0 (“worst imaginable health”) to 100 (“best imaginable health”). The respondent’s answers provide a description or profile of the respondent’s quality of life, and a weight or value can then be placed on each profile using an existing UK tariff derived from the general public [35–38]. The Eq-5D-5L will be completed at baseline and then at 14 and 26 weeks post-randomisation by the parent.

CHU-9D-proxy version (parent report on child): The CHU-9D is a paediatric measure of health-related quality of life, which allows the calculation of QALYs for use in cost-utility analysis. It includes nine dimensions (worried, sad, pain, tired, annoyed, schoolwork, sleep, daily routine, activities) each with five levels and has been validated in child and adolescent populations [39]. Parents will complete the proxy version at baseline and then at 14 and 26 weeks post-randomisation.

The Child Anxiety Impact Scale-parent report (CAIS-P) is the primary clinical outcome, but will be used also in the economic analyses. It captures the degree to which anxiety is interfering in the child and family’s life, measured at 26 weeks post-randomisation (see above).

Client Services Receipt Inventory (CSRI)—children’s version (parent report): A modified version of the CSRI [40] will be used to collect information on patient-level resource use for both children (parent-report on children) and parent (self-report). Parents will be asked to report use of health, social care and non-NHS services, medications, and parental time off work, school, and leisure activities at baseline (based on the prior 3 months) and then at 14 and 26 weeks post-randomisation. Parents will be provided with health diaries to facilitate recall of parent and child’s resource use.

Therapist Economic Logs (Supervision Logs and Treatment Logs): We will collect economic logs from therapists during the treatment phase (up to 26 weeks if applicable; in the C-TAU arm where there is no set end point to the treatment). Therapists in both trial arms will be asked to complete an economic log throughout treatment, to record all staff-time spent on treatment-related activities (e.g. training and supervision, preparation of sessions, administration, phone contact with parent, video-contact with child whenever applicable).

#### Treatment credibility and experience

Credibility and Expectation of Improvement Scale (CEI): Parents will be asked to complete the CEI to assess participant expectations and views regarding treatment credibility, after randomisation and prior to treatment commencing [41]. It consists of three items, rated on a scale from 0 ‘not at all’ to 10 ‘completely’, asking about how logical the treatment seems, confidence in its success at reducing their symptoms, and their likelihood to recommend the therapy to a friend with similar symptoms. This measure is administered after randomisation with reference to the allocated treatment arm.

An adapted version of the CEI will also be administered 14 weeks post-randomisation, to give a retrospective account of treatment credibility (i.e. the questions are reworded to be considered in light of having received treatment).

We have also adapted the CEI to evaluate therapists’ experiences of treatment within this trial. This comprises items referring to how logical they found the treatment, how comfortable they felt delivering the treatment, how prepared they felt, certainty in the success of the intervention, confidence recommending the treatment to other therapists, and likelihood of administering the treatment again. Completion of this is part of their delivery of the research study.

#### Adverse events reporting opportunity

Trial therapists will be asked to report any adverse events that they become aware of while working with families in either arm over the whole treatment period. We will also provide parents and children an opportunity to describe any negative impacts of participating in the study after completing the questionnaires at 14 and 26 weeks and, for participating parents, after completing the qualitative interview. So as not to ‘lead’ answers, we will enquire about both the positive and negative consequences of taking part in the treatment. The research team will regularly review responses to identify any responses that indicate the presence of an adverse event.

#### Measures routinely used to monitor outcomes in OSI

For the OSI+therapist support arm only, the OSI platform collects routine outcome measures and these will be used to help therapists to evaluate the progress of participants through treatment and to explore the trajectory of participant improvement across the course of treatment. The OSI platform routinely collects the CAIS-P, RCADS-p, SCAS-P8, and ORS as described above, and session rating scales and goal-based outcomes as described below:

Session Rating Scales (SRS): The SRS [42] assesses key dimensions of an effective therapeutic relationship and will be made available for completion at the end of each therapy session to get feedback from the parents so that any issues related to therapeutic alliances can be immediately identified and addressed within treatment. The SRS comprises four simple rating scales in which the parent rates their experience of the treatment session (with regard to relationship with the therapist, goals and topics, approach or method, and an overall rating). It uses the same visual analogue scale as the ORS. It has well-established reliability and validity [42, 43].

Goal-based outcomes (GBOs): This is a simple rating scale in which the parent rates on an 11-point scale (0–10) the extent to which their child has made progress towards up to three treatment goals [44]. Although this measure is now widely used in CAMHS (as part of the CYP IAPT initiative), its psychometric properties have not yet been established.

Routinely collected sessional measures will be used to explore the trajectory of change within the OSI+therapist support arm only to inform future developments of the programme. We will not be collecting routine outcome measures from the treatment as usual arm for comparative purposes as these will vary according to site-specific practice and treatment-specific requirements.

#### Qualitative interviews with parents and therapists

We will conduct qualitative interviews with 20–40 parents and therapists, purposively sampled on the basis of demographic characteristics to explore therapist and parents’ experiences of treating child anxiety problems in the current context. We have previously found that this sample size is sufficient for data saturation. Qualitative interviews will be conducted between 14 and 26 weeks after randomisation. Interviews will be conducted by a qualitative researcher and will be based on a topic guide (see Additional file 1), which will be developed and finalised following consultation with PPI representatives.

We will invite 10 parents and therapists from the first 20 participants to complete treatment to take part in the qualitative interviews and will review their demographics to identify characteristics that have not been represented using a grid developed specifically for this study to identify future participants to invite to take part (i.e. following a purposive sampling approach to ensure diversity among participants). We will initially invite participants to take part in the interview by email and will follow this up with telephone/text messages (up to three times). Interviews will be conducted remotely via Microsoft Teams (Teams) or over the telephone. Interviews will be audio recorded through Teams or an encrypted digital recorder. Recordings will be transcribed by the research team manually or via Teams and then checked/amended for accuracy. Information that could reveal the identity of a participant to other people will not be included in transcriptions. Transcripts will then be uploaded to NVivo [45] for analysis.

### Plans to promote participant retention and complete follow-up {18b}

Families will receive a £10 voucher as a thank you for completing extra questionnaires that they would not usually complete as part of routine care. Furthermore, parents or clinicians who take part in the additional qualitative interview will also receive a £20 voucher.

Shortly after being randomised to their treatment, participants will receive a telephone call welcoming them to the trial, explaining what is involved and answering any initial questions. To further promote participant engagement, families who are active in the study will be emailed a monthly parent bulletin, containing updates about the trial. There will also be a Twitter account that families will be invited to follow. In terms of completing follow-ups, there will be a scheduled series of emails, text messages, and telephone calls made to the families during the 1-month window that their 14-week and 26-week questionnaires are due. These will promote participant retention through reminding the families to complete the questionnaires and assist them with any difficulties they may be having.

Participants who discontinue treatment or deviate from protocol will be contacted by the research team and asked whether they would still be able to complete their follow-up questionnaires. In the event that the family are non-contactable, the research team will continue with the scheduled reminders unless the family state they wish to withdraw fully from the trial. The measures collected from these participants will be the same as per-protocol participants (outlined in Section 18a).

### Data management {19}

A study-specific Data Management Plan (DMP) will be developed for the Co-CAT trial outlining in detail the procedures that will be put in place to ensure that high-quality data are produced for statistical analysis.

On completion of the trial and data cleaning, the study documentation will be transferred to a secure, GCP-compliant archiving facility. Participants’ identifiable information will be kept for 6 months unless participants give permission for their information to be kept in order to be contacted about research after the study has finished. This excludes any research documents with personal information, such as consent forms, which will be held securely at the University of Oxford for 3 years after the end of the study. Qualitative interview transcripts will be retained securely to support publications of findings from the study until they are no longer required. Prior to database lock, the Data Manager and the Trial Statistician will undertake a dataset review. Digitised consent/assent forms and an electronic record of consent/assent completed online will be stored for 3 years after the study is complete and then securely destroyed. The linkage information will be permanently destroyed at the end of the study. The only exception will be that if participants (parents) agree to be approached for future research, we will retain the consent form as the basis for retention of details and future approach. Those contact details will be held securely, separately from the research data, and will be kept updated. Audio-recordings will be stored until recordings have been transcribed verbatim, and transcriptions thoroughly checked. This means that audio files will be destroyed by the end of the study.

#### Source data

Source documents are where data are first recorded, and from which participants’ Case Report Form (CRF) data are obtained. These include, but are not limited to,

- Parent-reported questionnaires
- Child-reported questionnaires
- Therapist-reported questionnaires
- Parent and therapist interviews

CRF entries will be considered source data if the CRF is the site of the original recording (e.g. there is no other written or electronic record of data). On all trial-specific documents, other than the signed consent, each participant will be referred to by the trial participant number/code, not by name.

#### Access to data

Direct access will be granted to authorised representatives from the Sponsor and host institution for monitoring and/or audit of the study to ensure compliance with regulations.

Access to personally identifiable data will be restricted on a need-to-know basis. This will include the PI, co-investigators, research staff involved in data collection, therapists, and clinical supervisors delivering the intervention.

Agreements will also be in place with external organisations (OSI hosting provider and software developer, a transcription service) who will be data processors and will require access to personal data collected in this study. Oxford University Third Party Security Assessments (TPSA) will be conducted for both organisations and they will be required to comply with the TPSA recommendation.

Anonymised trial data will be made available for open access on completion of the trial via the UK Data Service or another suitable repository. Qualitative interviews will not be shared in this way due to the difficulties in fully anonymising the data.

### Data recording and record keeping

All participants (parents, children, therapists) will be assigned a unique ID. A document that links the participant’s personally identifiable data with the participant ID will be stored separately from all other research data. Therapist records will refer to the study identifier of the parent from the participating family so that data can be linked. Sentry will be used to ensure that record of participant contact details (email, telephone number, address) is stored separately from other data. Sentry is an online secure data entry system developed in-house at PC-CTU and hosted at Oxford. It is designed to collect sensitive data, such as participant contact details, and securely retain them separate from a trial’s clinical data. Sentry can also act as a central participant portal to manage online eligibility, eConsent and ePRO—acting as an intermediary between the participant and the clinical databases. Sentry is accessed via a secure HTTPS connection and all stored sensitive data is encrypted at rest to AES-256 standards.

The main trial data will be entered directly into the participants’ CRFs in an electronic format by the participant or trial team (using REDCap database via Sentry). The use of REDCap is compliant with Good Clinical Practice and guidelines such as 21 CFR Part 11 via differentiated user roles and privileges, password and user authentication security, SSL encryption, and de-identification of Protected Health Information. Data will be hosted on network servers/drives which are maintained by University of Oxford MSD’s and are backed up every 24 h to and firewalls and authentication are in place to block any inappropriate access.

While all main trial data will be directly entered into electronic Case Report Forms (eCRFs), paper versions will be provided if access to the online CRF is not possible. In this case, the original copy of the CRF will be returned to the study team and a copy will be held at the research site. All CRFs (electronic or paper) will be date stamped upon receipt. A full pre-entry review and electronic data validation for all data entered into the clinical database will be provided by study-specific programmed checks (see Data Management and Checks section below). All paper data will be locked in secure cabinets and only the researchers will have access to the files. A separate database will be used to securely store all identifiable patient information required to contact patients and permit follow-up. Access to this information will be strictly on a need-to-know basis and databases will be password protected on a secure server.

Routine sessional treatment data will be captured within OSI (for the OSI+therapist support arm only) and this will be available to the research team in pseudo-anonymised form via the OSI researcher portal. The pseudo-anonymised data will be regularly exported from OSI in CSV form and saved on the study-restricted access OneDrive for merging (by study ID) with the main trial data at the point of statistical analysis. The OSI online platform is based in the European Economic Area and is compliant with NHS digital’s requirements for data and security. As noted above, OSI will be subject to a University of Oxford TPSA and will be required to comply with recommendations.

Qualitative interviews will be audio recorded using an encrypted digital audio recorder. Recordings will be held temporarily on these audio-recording devices for no more than 24 h before being securely transferred to a restricted access folder on the University of Oxford IT Network where they will be stored separately from all other research data in a restricted access OneDrive folder. The audio recording will be either (i) transcribed by a member of the research team (manually or using Teams) or (ii) sent securely via OneDrive to a transcriber with a contract with the university (and who has been subject to and found to comply with recommendations from a TPSA). The transcriber will be required to delete all audio files after returning the verbatim transcription to the research team.

Where Teams is used to transcribe interviews, the audio recording will be used to create a Teams transcript which will be automatically generated by Microsoft Teams in Nexus 365 STREAM, manually downloaded as a .vtt file to a temporary folder in the researcher’s computer, uploaded to Microsoft STREAM VTT cleaner to remove all coding from the transcript, and the bare text of the transcript then copied and pasted into a word document. The transcript will then be formatted, checked against the original audio, and names removed and saved to a secure restricted access OneDrive folder. The recording on MS STREAM and .vtt file will then be deleted. Transcripts will be de-identified.

### Data management and checks

The data management will be run in accordance with the University of Oxford Primary Care-Clinical Trials Unit (OC-CTU) Standard Operating Procedures, which are fully compliant with Good Clinical Practice (GCP). A PC-CTU data manager will be assigned to the study, as delegated by the CI, and will be responsible for overseeing the receiving, entering, cleaning, querying, analysing, and storing of all data that accrues from the study by designated persons.

#### Data entry

The data capture method used is Electronic Data Capture; therefore, single-pass entry is used rather than double-data entry. This is in accordance with the SOP PC-CTU_SOP_DM106_Data Entry.

#### Dataset reviews

· Full dataset reviews are conducted on a bi-monthly basis.
· AE/SAEs will be entered by the trial team by single data entry (a 100% SDV will be conducted to ensure accuracy at the end of the trial).

Date of birth check:

· The date of birth is entered by parents within the demographics questionnaire.
· These will be checked periodically (twice a week) to ensure the child meets the age criteria (child should be aged 5–12 years at intake)

Data quality rules (AE/SAE Forms):

· Looking at inconsistent data within the database
· Data quality rules are run once a month. These include (reverse checks):
  1. The date the form was completed is prior to the start date of the adverse event.
  2. The date the form was completed is in the future.
  3. The date the PI was made aware of the event is prior to the start date of the adverse event.
  4. The date the PI was made aware of the event is in the future.
  5. The date the adverse event was resolved is prior to the start date of the adverse event.
  6. The date the adverse event was resolved is in the future.
  7. It has been recorded that the participant outcome was death; however, it has also been recorded that the action taken was ‘Continued with study’.
  8. The date provided is in the future.

Listing checks:

· Report to ensure data is consistent across related forms.
· These are run once a month by the data manager

Edit Checks:

· There are 276 automatic edit checks that are built into the CDMS that fire upon submission of data.

Database rules and limits have been created to promote cleaner data follow entry. This means that variables can only accommodate certain values within a range to limit data entry mistakes. Limiting the free text box options and promoting the use of radio buttons and drop-down menus within the database results in cleaner data throughout.

#### Data storage and security

CDMS Back-up: Data stored on REDCap will be backed up on a nightly basis to the host server for 62 days. Back-up of the server file occurs nightly to two separate off-site storage facilities using University incremental back-up servers (IBIS). Each back-up is stored for approximately 6 months with one back-up retained for the 3 months preceding the start date of the retained daily back-ups.

Further details can be found in the Data Management Plan (available on request).

### Confidentiality {27}

The study will comply with the General Data Protection Regulation (GDPR) and Data Protection Act 2018, which require data to be de-identified as soon as it is practical to do so. The processing of the personal data of participants will be minimised by making use of a unique participant study number only on all study documents and any electronic database(s), with the exception of the CRF, where participant initials may be added. All documents will be stored securely and only accessible by study staff and authorised personnel. The study staff will safeguard the privacy of participants’ personal data.

Procedures will be compliant with:

- Data Protection Checklist https://researchsupport.admin.ox.ac.uk/policy/data/checklist
- Practical Considerations https://researchsupport.admin.ox.ac.uk/policy/data/practical

### Plans for collection, laboratory evaluation, and storage of biological specimens for genetic or molecular analysis in this trial/future use {33}

See above (26b) there will be no biological specimens collected.

## Statistical methods

### Statistical methods for primary and secondary outcomes {20a}

The statistical aspects of the study are summarised here. Details will be fully described in a statistical analysis plan (SAP). The SAP will be prepared by an independent statistician and finalised before any analysis takes place.

#### Description of the statistical methods

In accordance with CONSORT guidelines, we will record and report participant flow. Descriptive statistics of recruitment, drop-out, and completeness of interventions will be provided. Baseline variables will be presented by randomised group using frequencies (with percentages) for binary and categorical variables and means (and standard deviations) or medians (with lower and upper quartiles) for continuous variables. There will be no tests of statistical significance nor confidence intervals for differences between groups on any baseline variables.

##### Analysis of clinical outcomes

Analysis of the primary outcome will be performed using a generalised linear mixed effects model adjusting for stratification variables, and any baseline variables that are deemed to be highly prognostic of the outcome will be used to determine the treatment effect and 95% confidence interval. The mixed effect models will include the outcome as the response variable, time point, randomised group, and baseline score as fixed effects and a participant-specific random intercept. An interaction between time and randomised group will be fitted as a fixed effect to allow estimation of treatment effect at all time points. Non-inferiority is claimed if the lower limit of the 95% confidence interval around the standardised effect size is −0.33. A *P*-value for non-inferiority will also be calculated. A similar approach will be used for the other secondary outcomes.

##### Additional quantitative analyses

Treatment credibility, acceptability, and experience scores will be calculated and compared for both treatment groups, using simple mean comparisons. Change in child symptoms and functioning on a sessional basis will be plotted to explore the trajectory of change in the OSI arm.

##### Qualitative analysis

Transcribed interviews will be analysed using an inductive thematic analytic approach [46]. Rather than relying on a pre-existing coding or theoretical framework, codes and themes will be data-driven. A number of strategies will be employed to enhance the credibility and methodological rigour of the analysis, including the use of reflexive practices in supervisory discussion and presentation of the analysis to a small group of expert researchers and therapists.

#### Health economics analysis

In order for a new intervention to be widely adopted in the NHS, it is necessary to assess not only its clinical effectiveness but also its cost-effectiveness, namely whether the new intervention is good value for money compared with current practice. In other words, the performance of an economic evaluation alongside this RCT will allow us to establish whether OSI+therapist support is worth doing compared with C-TAU and whether we are satisfied that the health care resources required for OSI+therapist support to be made available to those who could benefit from it should be spent this way rather than some other way, i.e. C-TAU in this specific case. Therefore, the results of the economic evaluation will be invaluable to inform NHS decisions for treating child anxiety in such challenging circumstances and beyond.

The economic aspects of the study are summarised in this protocol. Details will be fully described in a health economics analysis plan (HEAP) [47], which will be finalised before any analysis takes place.

The economic evaluation will comprise cost-utility analysis (CUA) as primary analysis and cost-effectiveness analysis (CEA) as secondary analysis. They will be conducted from the NHS and personal services perspective (base-case analysis) as per NICE recommendations [38]. A wider societal perspective (including parental health care resource use and work productivity and school impacts) will be adopted in sensitivity analyses. We will follow best-practice guidelines for conducting and reporting economic evaluation analyses [38, 48]. Both an intention-to-treat and per-protocol approach will be adopted for primary and secondary analyses, as it is common in inferiority trials [49–51]. Missing data will be imputed by use of conditional mean imputation for missing values deemed highly deterministic (e.g. online/ face-to-face therapist contacts), and multiple imputation for other variables (e.g. GP consultations) under the assumption of missing at random. In the cost-utility analysis, the health outcomes will be quality-adjusted life years (QALYs) gained for the child (base-case analysis), and for the parent-child dyad (sensitivity analysis). QALYs will be derived from the collected measures CHU-9D [39] for the child, and EQ-5D-5L [35–38] for the parent.

In the secondary cost-effectiveness analyses, two outcomes will be considered, namely the primary clinical outcome (CAIS-P), and the percentage of school attendance. For each participant, components of treatment costs and other individual, family, and wider societal costs (as collected using the economic logs completed by therapists/ parents) will be computed by multiplying units of resource use by their unit costs and then summed to obtain a total cost per patient. Unit costs for health, social care, and other resources will be mainly derived from local and national sources [52] and estimated in line with best-practice. Costs will be expressed in pounds sterling at current prices. Given the short time-frame of the trial and follow-up, discounting will not be applied to costs or effects. The incremental costs and effects will be reported using incremental cost-effectiveness ratios (ICERs), where appropriate, and presenting cost-effectiveness acceptability curves [53]. Sensitivity analyses to explore uncertainty surrounding ICERs will be also conducted.

It is important to note that we originally planned to have two co-primary economic outcomes (cost-utility analysis (CUA) and cost-effectiveness analysis (CEA)); however, after initiating the trial, following consultation with expert colleagues, we decided that it would be better to have a single primary economic analysis, i.e. the CUA, and consider both the two CEAs as secondary. This is not only because the outcome of the CUA is what NICE prefers, but also to avoid not being able to come to a meaningful conclusion should the CUA and the CEA show contrasting results. This change was approved by the study ethical approval body on 20/9/2022.

### Interim analyses {21b}

#### Decision points

Due to the rapid nature of the trial, there will be no interim analyses.

#### Stopping rules

Due to the rapid nature of the trial, there is not an internal pilot and there are no formal stopping criteria.

### Methods for additional analyses (e.g. subgroup analyses) {20b}

No further analyses are specified at the moment but full details of all analyses will be provided in the Statistical Analysis Plan.

### Methods in analysis to handle protocol non-adherence and any statistical methods to handle missing data {20c}

#### Analysis populations

The primary analysis population is defined as all participants for whom data are available analysed according to the groups they were randomly allocated to, regardless of treatment compliance. They must have completed their assessment within 4 weeks of the 14-week and 26-week time points.

A per-protocol analysis will also be carried out excluding those who have deviated from the protocol. Compliance with protocol to be included in the per-protocol analysis will be defined as completing a minimum of the first 5 treatment sessions for participants in either arm (for OSI this is sessions 0, 1, 2, 3, and 4). Other analysis populations will be prespecified in the SAP.

#### Procedure for accounting for missing, unused, and spurious data

The availability of the primary outcome data will be summarised by randomised group. The mixed effects model implicitly accounts for data missing at random; however, the data missingness mechanism will be explored. Logistic regression models will explore any association between baseline characteristics and availability of the primary outcome. Missing primary outcome data will be reported overall and by randomised group. Covariates found to be predictive of missingness (*P* < 0.05) will be included in the analysis model in a sensitivity analysis of the primary outcome.

The response rates may differ between the intervention arms of the trial. A sensitivity analysis will be conducted that assumes data are missing not at random (MNAR).

#### Procedures for reporting any deviation(s) from the original statistical plan

Any deviations from the original statistical plan for the primary analysis will be discussed and agreed with the Trial Steering Committee and their agreement will be minuted. Deviations will be explicitly reported in subsequent trial reports.

#### Plans to give access to the full protocol, participant-level data, and statistical code {31c}

Anonymised trial data will be made available for open access on completion of the trial. Qualitative interviews will not be shared in this way due to the difficulties in fully anonymising the data. The Statistical Analysis Plan will be made available as a supplementary document to the main paper. Participant-level data and statistical code will be available upon request.

## Oversight and monitoring

### Composition of the coordinating centre and trial steering committee {5d}

The Trial Steering Committee (TSC) comprises relevant subject and methods experts and Patient and Public Involvement (PPI) representatives. We have agreed on terms of reference for the TSC. The TSC will meet every 4 months throughout the study or as necessary in agreement with TSC.

### Composition of the data monitoring committee, its role, and reporting structure {21a}

Recruitment to the trial will be rapid and no interim analyses are planned so a separate Data Monitoring and Ethics Committee will not be formed; however, we reserve the option to form one if the TSC deem it necessary at any point during the trial.

### Adverse event reporting and harms {22}

#### Definition of serious adverse events

A serious adverse events (SAE) is any untoward medical occurrence that:

- Results in death
- Is life-threatening
- Requires inpatient hospitalisation or prolongation of existing hospitalisation
- Results in persistent or significant disability/incapacity
- Consists of a congenital anomaly or birth defect

Other ‘important medical events’ may also be considered a serious adverse event when, based upon appropriate medical judgement, the event may jeopardise the participant and may require medical or surgical intervention to prevent one of the outcomes listed above.

NOTE: The term ‘life-threatening’ in the definition of ‘serious’ refers to an event in which the participant was at risk of death at the time of the event; it does not refer to an event which hypothetically might have caused death if it were more severe.

There is a very low risk of SAEs in the current trial; however, the following details a non-exhaustive list of potential SAEs and adverse vents (AE):

#### Potential serious adverse events (SAEs) (to parent/child)

1. Admission to a psychiatric hospital (parent/child).
2. Sectioned under the Mental Health Act.
3. Significant and sustained deterioration of pre-existing mental health condition that requires immediate intervention that cannot be accommodated within the treatment protocol (as determined in clinical supervision).
4. Diagnosis of new mental health condition.
5. Suicidal behaviour.
6. A serious safeguarding issue is revealed.

#### Potential serious adverse events (SAEs) not directly related to the trial and adverse events (AEs)

1. Children’s schooling or parent/guardians’ work is adversely affected (e.g. due to time spent in therapy or assessments encroaching on school or homework time).
2. One or more aspect of the therapy or assessment procedure induces unacceptable levels of distress for either the participant, their parent/guardian, or the therapist.
3. It becomes apparent that one or more of the exclusion criteria is met (or inclusion criteria not met) by the participant. [NB. This will be logged but the participant remains in treatment as long as clinically appropriate and retained in the intent to treat sample.]
4. A sustained and significant increase in detrimental behaviours (e.g. safety-seeking behaviours) as determined by any of the outcome measures collected throughout the study.
5. The emergence of new detrimental behaviours (e.g. self-harm).
6. Drop-out of treatment/request to change therapist (all routinely monitored for the presence of AEs).
7. A complaint is received from a participant, their parent/guardian, or the therapist referring to an actual or perceived adverse event as defined above.

The window for reporting SAEs and AEs will be:

(i) During the treatment phase based on therapist report
(ii) Up to the end of study based on parent report (i.e. up to the 26-week assessment or qualitative interview, whichever is later).

The 14-week and 26-week assessments will include questionnaires to monitor participants’ functioning and quality of life; therefore, information on some of the potential adverse events identified in this document will be routinely collected. Therapists will also be asked to indicate the presence of an SAE or AE that arises during the course of treatment. Further investigation will be made by the clinical team and the PI and the SAE/AE procedures will be followed where applicable. Specifically, the clinical team and the PI will assess the frequency and severity of the adverse event(s) and determine whether the participant should be withdrawn from the trial. The decision of the clinical team will be followed in the event that a consensus is not reached. Participants will also be given the opportunity to report adverse events anonymously after the parent and child complete questionnaires at 14 and 26 weeks and in person (remotely) after the qualitative interviews (for the parents). The research team will regularly review responses to identify any responses that indicate the presence of an adverse event and will report summaries of these responses to the Trial Steering Committee for review.

#### Reporting procedures for adverse events/serious adverse events

All AEs and SAEs will be recorded, logged, and monitored by the PI and TMG. If an AE is reported more than once for a participant, or more than three times during the study, it will be treated as an SAE.

A serious adverse event (SAE) occurring to a participant will be reported to the REC that gave the favourable opinion of the study where in the opinion of the Chief Investigator the event was ‘related’ (resulted from administration of any of the research procedures) and ‘unexpected’ in relation to those procedures. Reports of related and unexpected SAEs will be submitted within 15 working days of the Chief Investigator becoming aware of the event, using the HRA report of serious adverse event form.

In the event that a complaint is received from a participating parent or child, the therapists, or their managers, the CI will attempt to resolve the issue as far as is possible. If this is not possible, and the issue remains unacceptable to participants, formal complaints will be logged and dealt with by the sponsor’s representative in liaison with the CI. Those indicating an AE or SAE will be logged accordingly.

### Frequency and plans for auditing trial conduct {23}

The study may be monitored, or audited in accordance with the current approved protocol, GCP, relevant regulations, and standard operating procedures. In addition, a risk assessment will be carried out and provided to the CTRG before the study opens and will be reviewed as necessary over the course of the study to reflect significant changes to the protocol or outcomes of monitoring activities (e.g. monitoring of adverse events).

### Plans for communicating important protocol amendments to relevant parties (e.g. trial participants, ethical committees) {25}

Following Sponsor approval, the protocol, informed consent form, participant information sheet, and any proposed advertising material will be submitted to an appropriate Research Ethics Committee (REC), HRA, and host institutions for written approval. The Investigator will submit and, where necessary, obtain approval from the above parties for all substantial amendments to the original approved documents. For protocol modifications, we will first seek approval from the TSC and then apply for approvals as required.

### Dissemination plans {31a}

A summary of the study findings will be circulated at the end of the study to all participating clinical teams and families. Peer-reviewed publications will be produced to reach a wider audience.

## Discussion

This study has been designed to be a pragmatic trial embedded in the services which would be the typical users of this type of brief intervention for child anxiety problems. This is a definite strength of the trial design, as it maximises the likelihood that the results will reflect the outcomes that could be expected in clinical practice outside of a trial. Designing the trial to be almost entirely managed using online platforms will enable us to have large numbers of trial sites spread across the UK, and to involve clinical teams who have previously not taken part in the research.

If non-inferiority is found, the research will provide the following: (1) a solution for efficient psychological treatment for child anxiety disorders while social distancing (for the current context and future pandemics); (2) an efficient means of treatment delivery as ‘normal service’ resumes to enable CAMHS to cope with the anticipated increase in referrals when social distancing measures are relaxed and schools re-open; and (3) a demonstration of rapid, high-quality evaluation and application of online interventions within NHS CAMHS to drive forward much-needed further digital innovation and evaluation in CAMHS settings. The primary beneficiaries will be children with anxiety problems and their families, NHS CAMHS teams, and commissioners who will access a potentially effective, cost-effective, and efficient treatment for child anxiety problems.

### Limitations and barriers

Conducting a trial during the COVID pandemic is challenging. From a structural perspective, support during this period for clinical teams taking part in the trial may not always be available, and also an increased, and in many cases more severe, number of referrals is likely to put significant pressures on clinical teams. We are reliant on parent and child reports of treatment outcomes and do not have the capacity to conduct systematic clinical assessments within the trial procedures. We are also reliant on clinical teams and families to confirm inclusion/exclusion criteria; however, this does mean that the participants reflect families who would likely be offered the novel intervention within routine clinical services.

### Trial status

Protocol V2.3, 15.07.2022

Recruitment start date: 05/12/2020

End date for recruitment 03/08/2022

## Supplementary Information

**Additional file 1.** Indicative topic guides.

**Additional file 2.** FMWOfferDoc_17417979. Original funder approval (MRC).

**Additional file 3.** NIHR204435 Intend to Fund letter. Original funder approval (NIHR).

**Additional file 4.** Original ethics approval.

## Data Availability

The final trial dataset will be accessed by the trial statisticians and health economist. There are no contractual agreements in place that limit access for investigators. Anonymised quantitative trial data will be made available for open access on completion of the trial.

## Abbreviations

AE: Adverse event
AES-256: Advanced Encryption Standard - 256
AHSN: Academic Health Science Networks
AnDY: Anxiety and Depression in Young People Research Clinic (University of Reading)
CAIS-C: Child Anxiety Impact Scale—Child
CAIS-P: Child Anxiety Impact Scale—Parent
CAMHS: Child and Adolescent Mental Health Services
CBT: Cognitive Behavioural Therapy
CEA: Cost-effectiveness analysis
CEI: Credibility and Expectation of Improvement Scale
CHU-9D: Child Health Utility
CI: Chief Investigator
Co-CAT: Child Anxiety Treatment in the context of COVID-19
Co-SPACE: COVID-19: Supporting Parents, Adolescents and Children during Epidemics
COVID-19: Coronavirus disease 2019
CRF: Case Report Form
CSRI: Client Services Receipt Inventory
C-TAU: Treatment as Usual in the context of COVID-19
CTRG: Clinical Trials & Research Governance, University of Oxford
CUA: Cost-utility analysis
CYP: Children and young people
DHSC: Department of Health and Social Care
DMEC: Data Monitoring and Ethics Committee
DMP: Data Management Plan
eCRF: Electronic Case Report Form
EQ-5D-5L: European Quality of Life-5 Dimension 5 - Level (adult quality of life instrument)
ePRO: Electronic Patient-Reported Outcome
GBO: Goal-based outcomes—goal progress chart
GCP: Good Clinical Practice
GDPR: General Data Protection Regulation
GP: General Practitioner
HRA: Health Research Authority
HTTPS: Hypertext Transfer Protocol Secure
IAPT: Improving Access to Psychological Therapies
ICER: Incremental Cost-Effectiveness Ratios
ICF: Informed consent form
ICMJE: International Committee of Medical Journal Editors guidelines
IRAS: Integrated Research Application System
IP: Internet Protocol
ISF: Investigator Site File
ISRCTN: International Standard Randomised Controlled Trials Number
ITT: Intention to treat
MRC: Medical Research Council
MSD: Medical Science Division
NHS: National Health Service
NICE: The National Institute for Health and Care Excellence
OID: Organisational Information Document
OSI: Online Support and Intervention (for child anxiety)
PC-CTU: Primary Care Clinical Trials Unit
PI: Principal Investigator
PIL: Participant/Patient Information Leaflet
PPI: Patient and Public Involvement
Q&A: Question and answer
QALYS: Quality-adjust life years
RCADS-C/P: Revised Children’s Anxiety and Depression Scale – Child/ Parent
RCT: Randomised controlled trial
R&D: NHS Trust R&D Department
REC: Research Ethics Committee
REDCap: Research Electronic Data Capture
RES: Research Ethics Service
SAE: Serious adverse event
SAP: Statistical analysis plan
SCAS-P8: Brief Spence Children’s Anxiety Scale-Parent Version
SDQ-p: Strengths and Difficulties Questionnaire- parent report
SOE: Schedule of events
SOP: Standard Operating Procedure
SPIRIT: Standard Protocol Items: Recommendations for Interventional Trials
SRS: Session Rating Scale
SSL: Secure Socket Layer Encryption
TAU: Treatment as usual
TMG: Trial Management Group
TSC: Trial Steering Committee
UKRI: UK Research and Innovation
VAS: Visual Analogue Scale
