# Supplementary material for: A randomised controlled trial to compare clinical and cost-effectiveness of an online parent-led treatment for child anxiety problems with usual care in the context of COVID-19 delivered in Child and Adolescent Mental Health Services in the UK (Co-CAT): a study protocol for a randomised controlled trial
Source: Trials. 2022 Nov 16;23:942. doi: 10.1186/s13063-022-06833-5 (PMC9667839; doi:10.1186/s13063-022-06833-5)
Supplement: Supplementary file 1 — Additional file 1. Indicative topic guides. [file 13063_2022_6833_MOESM1_ESM.zip › Co-CAT Indicative topic guide - Clinician V1.0 21.08.2020.R2.pdf]

Co-CAT: Indicative topic-guide for post-treatment qualitative interviews with therapists

Introduction

Interested in talking about your experience of delivering the treatment – the training, putting it into practice, the supervision and generally being involved in the study.

- What were your initial thoughts about the process of being asked to take part? *[Prompts: leaflets; positives/negatives/impact on their time, standard of care]*
- What was your understanding of the aims of the study?
- How did you feel about being allocated to deliver the [‘online’ group or the ‘treatment as usual’ group? *[Prompts: any concerns, positive/negative expectations]*

Prior to getting involved in the trial

- Can you tell me about your most recent role?
- How would you describe the way that you have usually worked with CAMHS clients? And children with anxiety problems in particular?

Getting involved

- Can you tell me about how you came to get involved in this study?
- What did you expect?
- Was there anything that particularly helped you to decide?
- Was there anything that made it difficult to make this decision?
- Was there anything that might have made a difference?

Therapists who delivered the online intervention group

Describing and evaluating the treatment

i) OSI training

- How did you find the training? [*Prompt: What was your experience of the training video/manual/Q&A? How much did you use the training? How did you use/draw upon the resources (e.g. only accessed at beginning, or accessed throughout)?]*
- What were the most and least helpful parts?
- If you could add something to it, what would it be?

ii) online programme

- Can you tell me a bit about the website
    - How did you find using the website?
    - Is there anything you particularly liked/didn't like about the website? / used a lot/ didn't use?
    - Did you experience any problems with the website?
    - How could the website be improved?
- I'm interested to hear your thoughts about different parts of the website *[prompt for feedback on videos, animations, audios, therapy session agenda, interactive elements]*
- What did you think about the game for your child? Did families you worked with use it? *[prompts– how much did your child use the game? ways it was helpful/not helpful]*

iii) telephone sessions

- Can you tell me a bit about delivering the telephone sessions? [*prompts*: *what was helpful/unhelpful, easy/difficult, best thing, worse thing*?]

iii) involving or talking to others

- Can you tell me how you think your client(s) found using the online programme?
- What did other members of your clinical team think about using an online programme to deliver therapy?
- Would you recommend using OSI to your colleagues? [*prompt for reasons why/why not, circumstances would recommend/would not recommend*]

iii) overall – since treatment ended

- What was your overall impression of using the online system as a part of therapy?
- How did this online treatment differ to your usual (recent) practice delivered in the context of COVID-19?

Therapists who delivered treatment as usual

Describing and evaluating the treatment

- Can you tell me a bit about the treatment(s) you delivered? [*prompts*: *who attended, format, number and content of sessions, resources used in sessions, clinician*]
- What kinds of things did you do with your client(s)? (What did that involve? Where did you do the work? How did it go? What was it like for you? How do you think the client found it? What did you learn?).
- How did delivering this treatment in the context of COVID-19 compare to what you would normally do?
- What helped you to deliver the treatment, what got in the way, what might have made a difference, but wasn't available to you?

All Therapists

Evaluating acceptability of the research process and context

- What did you think about being part of a research trial? Did you notice anything about that?
- Is there anything you found challenging about being part of a research trial? [prompt: what worked well/ less well, was *easy/difficult, best thing, worse thing*?]
- What is your understanding of the research aspects of the study compared to the clinical aspects?
- How did you find incorporating this extra load (if there was any)?
- Is there anything that would have made this easier?
- If a colleague or friend was interested in taking on a trial therapist role, what would you tell them about this way of working?
