# Supplementary material for: A randomised controlled trial to compare clinical and cost-effectiveness of an online parent-led treatment for child anxiety problems with usual care in the context of COVID-19 delivered in Child and Adolescent Mental Health Services in the UK (Co-CAT): a study protocol for a randomised controlled trial
Source: Trials. 2022 Nov 16;23:942. doi: 10.1186/s13063-022-06833-5 (PMC9667839; doi:10.1186/s13063-022-06833-5)
Supplement: Supplementary file 1 — Additional file 1. Indicative topic guides. [file 13063_2022_6833_MOESM1_ESM.zip › Co-CAT Indicative topic guide - Parent V1.0 21.08.2020R2.pdf]

Co-CAT: Indicative topic-guide for post-treatment qualitative interviews with parents

All parents

Consenting to take part
- Can you tell me about the journey from being referred to CAMHS to getting help? *[Prompts: waiting for help, emotions]*
- Can you tell me a little bit about what you remember about someone talking to you about the research for the first time?
- What were your initial thoughts about the process of being asked to take part? *[Prompts: leaflets; positives/negatives/impact on their care in CAMHS – quality of treatment, getting further help afterwards]*
- What was your understanding of the aims of the study?
- How did you feel about being allocated to the ['online' group or the 'treatment as usual' group? *[Prompts: any concerns, positive/negative expectations]*

Parents in the online intervention group only

1. Describing and evaluating the treatment

i) online programme
- Can you tell me a bit about the website
    - How did you find it using the website?
    - Is there anything you particularly liked/didn't like about the website? / used a lot/ didn't use?
    - Did you experience any problems with the website?
    - How could the website be improved?
- I'm interested to hear your thoughts about different parts of the website *[prompt for feedback on videos, animations, audios, therapy session agenda, interactive elements]*
- What did you think about the game for your child? Did they use it? *[prompts– how much did your child use the game? ways it was helpful/not helpful]*

ii) telephone sessions
- Can you tell me a bit about the telephone sessions with the therapist
    - What kinds of things did you talk about?
    - How did you find talking to the therapist on phone? [*prompts*: *what was helpful/unhelpful, easy/difficult, best thing, worse thing*?]
- Can you think of times when you drew upon what you talked about in telephone session? [*prompt for any examples of changes in parent/child feelings, thoughts, behaviour*].

iii) involving or talking to others
- Did anyone else do the online sessions with you? How was that?

- Did you tell other people about you doing the online programme? What did they make of it?
- Did you tell other people about the telephone sessions with a therapist? What did they make of it?

iii) overall – since treatment ended

- How are things now? Do you feel the treatment was helpful/not helpful? What difference do you think the treatment made?
    - Have you been using the website since your regular therapy sessions have finished? [*prompt for amount of use, reasons use/do not use, helpful/unhelpful since end of treatment*]
    - How have you got on putting things into practice since your telephone sessions ended?

Parents in the treatment as usual group only

1. Describing and evaluating the treatment

- Can you tell me a bit about the treatment? [*prompts*: *who attended, format, number and content of sessions, resources used in sessions, clinician*]
    - What did you think of the treatment? [*prompts*: *what was helpful/unhelpful, easy/difficult, best thing, worse thing*?]
    - How could the treatment be improved? [*prompts*: *who attended, format, number and content of sessions, clinician*]
- How are things now? What difference do you think the treatment made?
    - Have you/child continued to put anything from the treatment into practice?

All parents

Evaluating acceptability of the treatment and research process

- Would you recommend the treatment that you had to a friend? [*prompt for reasons why/why not, circumstances would recommend/would not recommend*]
- What did you think about the questionnaires that you were asked to complete? Were any of them asking particularly relevant questions or particularly irrelevant questions for you?
- What did you think about being part of a research trial? Did you notice anything about that?
