# Supplementary material for: A randomised controlled trial to compare clinical and cost-effectiveness of an online parent-led treatment for child anxiety problems with usual care in the context of COVID-19 delivered in Child and Adolescent Mental Health Services in the UK (Co-CAT): a study protocol for a randomised controlled trial
Source: Trials. 2022 Nov 16;23:942. doi: 10.1186/s13063-022-06833-5 (PMC9667839; doi:10.1186/s13063-022-06833-5)
Supplement: Supplementary file 2 — Additional file 2. FMWOfferDoc_17417979. Original funder approval (MRC). [file 13063_2022_6833_MOESM2_ESM.pdf]

Head of Department
Central Admin - Research Services
University of Oxford
Wellington Square
Oxford United Kingdom
OX1 2JD

Grant Ref: MR/V028510/1

Date: 18 August 2020

Dear Head of Department

GRANT OFFER: Research Grant, Research Grants
GRANT TITLE: Enabling Child and Adolescent Mental Health Services (CAMHS) to provide efficient remote treatment for child anxiety problems in the COVID-19 context

The MRC is offering a grant towards the cost of the above project, subject to the terms and conditions set out below.

Return of the 'Offer Acceptance' will be taken as acceptance of the grant on the terms stated. If you are unable to accept the grant you should return a 'Decline' confirmation as soon as possible. Upon receipt of the 'Offer Acceptance' a 'Start Confirmation' request will be issued.

Grants are cash limited and expenditure against the grant must not exceed the value awarded apart for reasons stated in the standard terms and conditions.

Please note copies of this letter have not been sent to the grant holder and co-investigators (as appropriate); it is your responsibility to distribute copies as is necessary.

Yours faithfully

Grants Pre Award Team
RCUK Grants
A service provided on behalf of MRC

Organisation: University of Oxford

Grant Holder: Prof. Cathy Creswell

Grant Title: Enabling Child and Adolescent Mental Health Services (CAMHS) to provide efficient remote treatment for child anxiety problems in the COVID-19 context
Starts: 28 August 2020 **Ends:** 27 August 2021 **Duration:** 12

GRANT VALUE

Funds Awarded

	Authorised FEC (£)			RC Contribution (£)			
	net	Indexation	Total	net	Indexation	Total	% FEC
DI - Staff	199,553	1,616	201,169	159,642	1,293	160,935	80
DI - T&S	2,000	16	2,016	1,600	13	1,613	80
DI - Other Costs	32,590	264	32,854	26,072	211	26,283	80
DA - Investigators	33,893	275	34,167	27,114	220	27,334	80
DA - Estate Costs	80,817	655	81,471	64,653	524	65,177	80
DA - Other Directly Allocated	2,957	24	2,981	2,366	19	2,385	80
Indirect - Indirect Costs	258,398	2,093	260,491	206,719	1,674	208,393	80
**Total Value of Award**	**610,208**	**4,943**	**615,151**	**488,166**	**3,954**	**492,121**	

Cost of Access to Facilities 0
(Funds not awarded to Grant Holding Organisation)

STAFF

Staff Summary

	Authorised FEC net	RC Contribution net	Number Of Staff Months
Investigator	43,703	34,963	5
Researcher	189,742	151,794	51

Staff and DI Investigator Details

Start Date	End Date	Duration	FTE Percent	Name or Post Identifier	Summary Fund Heading	Authorised Cost (Excluding Indexation)
28 August 2020	27 August 2021	12	8	Dr M Violato	Directly Incurred	5388.32
28 August 2020	27 August 2021	12	5	Associate Professor L Yu	Directly Incurred	4422.24
28 August 2020	27 August 2021	12	30	Nicola Williams	Directly Incurred	20882.74
1 January 2021	31 July 2021	7	100	PDRA	Directly Incurred	25986.57
28 August 2020	27 November 2020	3	100	Data Manager	Directly Incurred	11830.34
28 August 2020	27 August 2021	12	60	Health Economist	Directly Incurred	29261.29
28 August 2020	27 August 2021	12	100	PGRA 1	Directly Incurred	39469.54
28 August 2020	27 August 2021	12	50	Mrs Lucy Taylor	Directly Incurred	22842.30
28 August 2020	27 August 2021	12	100	PGRA 2	Directly Incurred	39469.54

DA Investigator Details

Average Hours/week	Name or Post Identifier
3.8	Prof. C Creswell
3.8	Dr C Hill
1.9	Dr V Raymont
1.9	Dr P Waite

EQUIPMENT DETAILS

Description	Delivery Date	Country Of Origin	Total Value

FACILITY AND SERVICE DETAILS

Facility	Cost of Access	Number of Units

PROJECT PARTNERS

Organisation	Department	Last Name	First Name	In Kind Value (£)	Monetary Value (£)

GRANT ADDITIONAL INFORMATION

GRANT CONDITIONS

CALL CONDITIONS

In addition to the UKRI standard T&Cs included with this award, the following terms apply:

1. The start date of this Grant must be no later than four weeks of the award notification email date, therefore this award will have a fixed latest start date applied. The standard three months start period rules stipulated in the 'Standard Terms and Condition of Grant RGC 5' are therefore not applicable to this project.

2. As this is a rapid response project, Grant extensions will only be considered under exceptional circumstances (in line with the Equality Act 2010) and will require agreement by Us on a case-by-case basis. The Research Organisation remains responsible for compliance with the terms of the Equality Act 2010 including any subsequent amendments introduced while work is in progress; and for ensuring that the expectations set out in RGC 3.4 are met.

3. UKRI reserves the right to ask projects to become part of wider consortia or join with already existing efforts.

4. In addition to RCG 12, Grant Holders undertaking work relevant to coronavirus public health emergency are required to share their research data and findings as rapidly and widely as possible, including with public health and research communities and the World Health Organization in accordance with the statement on sharing research relevant to Covid-19 set out here: https: //wellcome.ac.uk/coronavirus-covid-19/open-data

Grant holders should therefore ensure:

i) research findings are made available via preprint servers such as https://arxiv.org/, https://www.biorxiv.org/, https://psyarxiv.com/, https://www.medrxiv.org/ or https://socopen.org/welcome/, as soon possible and in any event before journal publication, or via platforms that make papers openly accessible before peer review.

ii) research findings that are submitted to journals are made openly available at the time of publication and released under the Creative Commons Attribution license (CCBY), see https://creativecommons.org/licenses/.

iii) all submissions (to platforms, preprint servers and journals) include clear statements regarding the availability of underlying data and/or software/code

iv) interim and final data is shared as rapidly and openly as possible - as soon as it is appropriately quality assured, in line with any ethics requirements, disciplinary good practice and irrespective of the timing of journal publication.

a. It is acknowledged that not all data can be open and often access needs to be managed, however in all cases the discovery of data is a priority. Data should be discoverable via data catalogues like the https://www.clinicalstudydatarequest.com/ and access conditions clearly laid out.

b. Examples of suitable data repositories for data sharing and access include, UKRI-funded data services such as the EMBL-EBI at https://www.covid19dataportal.org/submit-data, the UK Essex Data Archive at https://www.ukdataservice.ac.uk/deposit-data/how-to.aspx or the dedicated Coronavirus repository at OpenAIRE that is hosted at https://zenodo.org/communities/covid-19?page=1&size=20. Other repositories can be found on registries such as https://fairsharing.org/collection/COVID19Resources or the https://www.re3data.org/.

v) Software, analysis scripts or modelling codes created as part of the work under this funding are shared as rapidly and openly as possible.

a. Software, analysis scripts or modelling codes must be made available with a suitable licence via a platform that supports access and versioning. Suitable software platforms include: https://bitbucket.org/, https://circleci.com/integrations/github/ or https://about.gitlab.com/. If possible, an open source licence should be used, suitable licences that are available can be found at https://choosealicense.com/, however where software or scripts have a third-party dependency this must be respected.

b. To support longer term access software and code should be deposited in a repository that supports persistence, many data repositories, such as those referred to at iv) can provide this function or there are others dedicated to software such as https://www.softwareheritage.org/. These repositories support persistence more fully than the software platforms listed above.

The MRC Regulatory Support Centre (https://mrc.ukri.org/research/facilities-and-resources-for-researchers/regulatory-support-centre/) can help you with regulatory and ethics requirements, for research involving human participants, samples or data. Email: rsc@mrc.ukri.org

In addition to the UKRI standard, MRC additional T&Cs and other conditions included, this award also includes the following additional conditions for awards funded under the MRC (Invite Only) COVID-19 Rapid Response Rolling Call:

The start date of the award can be no later than four weeks of the award notification email.

Please note that due to the fixed start date, the standard starting period rules outlined in UKRI Terms and Conditions RGC5 do not apply to this project.

The investigators must acknowledge the UKRI (MRC), the DHSC (NIHR) support in any publications or events associated with this grant.

As this is a rapid response, project timeline grant extensions will not be considered.

In addition to the data sharing terms and conditions at AC10 researchers undertaking work relevant to public health emergencies are required to set in place mechanisms to share quality-assured interim and final data as rapidly and widely as possible, including with public health and research communities and the World Health Organization in accordance with the Joint statement on sharing research data and findings relevant to the novel coronavirus outbreak.

Please note that this award is currently in confidence pending formal announcement of the funding and should not be shared more widely until the UKRI/DHSC press release has been issued.

Ethical requirements for international grants:

It is the responsibility of the Principal Investigator and the Research Organisation to ensure that appropriate ethical approval is granted for this study and adhered to, and that no research requiring ethical approval is initiated until it has been granted.

MRC current policy for research involving humans, http://www.mrc.ac.uk/news- events/publications/research-involving-human-participants-in-developing- societies/ is that for research to be undertaken overseas, both local and UK ethical approval is required.

For clinical studies involving human participants and/or patients appropriate consent must be obtained.

For grants that include the use of animals, the guidance http://www.mrc.ac.uk/news-events/publications/responsibility-in-the-use-of-animals-in-research/ must be adhered to, and in particular: 'When collaborating with other laboratories, or where animal facilities are provided by third parties, researchers and the local ethics committee in the UK should satisfy themselves that welfare standards consistent with the principles of UK legislation (e.g. the ASPA) and set out in this guidance are applied and maintained.'

The Principal Investigator/ Research Organisation must be prepared to furnish the MRC with a copy of the ethical approval, and any correspondence with the committees, if requested by the Council. The principal investigator must notify the MRC if a regulator or a research ethics committee requires amendments that substantially affect the research question, methodology or costs to the extent that the project is no longer the same as that approved for funding by the MRC.

In Addition to RGC 12.4, the Grant Holder should inform the UKRI communications team by e-mail (press@ukri.org) at least 24

hours in advance before publication (either via a peer-reviewed publication, a pre-print or a press announcement) of the results of research funded by this grant.

RESEARCH COUNCIL CONDITIONS

SCHEME CONDITIONS

UK RESEARCH AND INNOVATION fEC GRANTS

STANDARD TERMS AND CONDITIONS OF GRANT

Introduction

UK Research and Innovation fEC Grants Standard Terms and Conditions of Grant
The Standard Terms and Conditions of Grant apply to Research Grants and Fellowships, costed and funded on a Full Economic Costs basis(fEC) and calculated according to the Transparent Approach to Costing (TRAC) or an equivalent methodology, awarded by the following seven UK Research and Innovation (UKRI) Councils:

Arts and Humanities Research Council (AHRC)
Biotechnology and Biological Sciences Research Council (BBSRC)
Economic and Social Research Council (ESRC)
Engineering and Physical Sciences Research Council (EPSRC)
Medical Research Council (MRC)
Natural Environment Research Council (NERC)
Science and Technology Facilities Council (STFC)

Application of Standard Terms and Conditions of Grant
In these Standard Terms and Conditions of Grant, the words "We", "Our" or "Us" refer to the relevant Council of UKRI awarding the Grant and "You" or "Your" refer to the Research Organisation in receipt of the Grant. Other key terms used in these Standard Terms and Conditions of Grant are set out in the Definitions attached at Annex A.

These Standard Terms and Conditions of Grant, together with any applicable Specific Terms and Conditions of Grant required by an individual Council of UKRI comprise the Grant Terms and Conditions on which UKRI awards the Grant to the Research Organisation. Specific Terms and Conditions of Grant will be set out in the Grant Offer Letter.

These Grant Terms and Conditions should be read in conjunction with the sources outlined in Annex B, in the event of any conflict the terms of these Conditions should prevail.

Use of Grant Proposal Information
UK Research and Innovation (UKRI) handles all personal data in accordance with current UK data protection legislation and the EU General Data Protection Regulation (GDPR) where appropriate.

It is the responsibility of the Research Organisation to ensure that both students it funds from UKRI funding and individuals who receive grant funding, or who are later involved in the award, are made aware of how personal data may be used by both UKRI and the Research Organisation. This includes information relating to groups such as students, supervisors, project partners, investigators, named researchers and support staff.

To meet UKRI's obligations for public accountability and the dissemination of information, contents of funded research proposals will also be made available on the Councils' websites and other publicly available sources. As a condition of funding, UKRI may use the data to publish information on awards made. We may also share information with third parties to support, for example, open access publication and reporting outcomes via Researchfish. This includes data submitted through Je-S Student Details (SD).

UKRI is also subject to the UK Freedom of Information Act (2000) and the Environmental Information Regulations (2004) and may be required to release grant information on request, subject to appropriate exemptions.

Further information is provided by the UKRI Use of grant proposal information addendum (www.ukri.org/files/funding/tcs/grants-addendum-pdf/) and via the UKRI Privacy Notice (www.ukri.org/privacy-notice/).

Standard Terms and Conditions of Grant

RGC 1 Variation to Terms and Conditions
UKRI reserves the right to amend and vary these Standard Terms and Conditions of Grant and any Specific Terms and Conditions of Grant or applicable policies at any time. The latest version of the Standard Terms and Conditions of Grant apply to all Grants with immediate effect and supersede any previous Standard Terms and Conditions under which a Grant was awarded unless otherwise stated. However, any Specific Terms and Conditions of Grant will still apply. Additional costs incurred as a direct result of changes made to Our Terms and Conditions should be managed within the Grant cash limit. Where the cash limit is exceeded solely due to costs incurred as a result of changes made to Our Terms and Conditions, a case can be made to Us for additional funds on an exceptional basis. The latest version of the Standard Terms and Conditions of Grant are available on the UKRI website at: https://www.ukri.org/funding/information-for-award-holders/grant-terms-and-conditions/

RGC 2 Accountability & Responsibilities of the Research Organisation
RGC 2.1 You are responsible for ensuring that the Project carried out by You, the Grant Holder and any Research Workers or other Third Parties, comply with these Standard Terms and Conditions of Grant and any Specific Terms and Conditions of Grant.

RGC 2.2 You must ensure that the Project is carried out in accordance with all applicable ethical, legal and regulatory requirements including but not limited to relevant provisions of the General Data Protection Regulation, the Data Protection Act 2018, the Bribery Act 2010, the Fraud Act 2006, the Equality Act 2010 and the Modern Slavery Act 2015.

RGC 2.3 You must ensure that Your use of the Grant complies with European Union State Aid(1) law. Where You are informed or You are aware that Your use of the Grant counts as De Minimis Aid(2), the financial limit must not be breached. All other use of the Grant which counts as Aid must fall under the General Block Exemption Regulation(3), it is Your responsibility to inform Us of any State Aid derived throughout the Grant Period. You acknowledge that if You breach State Aid law, UKRI may be required to recover some or all Grant funding, together with interest. For further information please refer to the Department for Business Innovation and Skills: The State Aid Manual.

(1) - Including but not limited to Articles 107 to 109 of the Treaty on the Functioning of the European Union, the General Block Exemption Regulation and any Enabling Regulation, as amended from time to time
(2) - Commission Regulation (EU) No 1407/2013
(3) - Commission Regulation EU No. 651/2014

RGC 2.4 You are accountable for the conduct of the Project including the conduct of the research, the use of public funds and the proper financial management of the Grant in accordance with these Standard Terms and Conditions of Grant and any Specific Terms and Conditions of Grant, whether the Project is carried out by You or the Grant Holder, Research Workers or other Third Party.

RGC 2.5 You must ensure that the Grant is spent in a way that is consistent with the purpose and conditions set out in the Offer Letter.

RGC 2.6 You must carry out appropriate due diligence on any Third Parties used to deliver any part of the Project and shall ensure in particular, that such Third Parties comply with these Standard Terms and Conditions of Grant and any Specific Terms and Conditions of Grant. At UKRI's request, You must provide details of expenditure of the Grant by any Third Party. Where all, or part, of the Project is carried out by Third Parties based overseas, You must follow the UKRI International Due Diligence Guidance: https://www.ukri.org/files/funding/due-diligence-guidance-for-ukros-pdf/

RGC 2.7 You must ensure that any part of the Full Economic Cost not funded by the Grant is committed to the Project before it starts.

RGC 2.8 You must have adequate business continuity plans in place to ensure minimum operational interruptions to the Project.

RGC 2.9 In order to foster a research culture which values, recognises and supports public engagement, You must adopt the principles, standards and good practice for public engagement with research set out in the 2010 Concordat for Engaging the Public with Research: https://www.ukri.org/public-engagement/research-council-partners-and-public-engagement-with-research/embedding-public-engagement/

RGC 2.10 You must notify UKRI of any changes to Your constitution, legal for, membership structure (if applicable) or ownership, including those that might affect Your eligibility to hold the Grant, or to deliver the Project or any other changes which affect Your ability to comply with the Grant Terms and Conditions.

RGC 2.11 You must ensure that the requirements of the Employing Organisation under the UK Policy Framework for Health and Social Care Research (or equivalent) are met for research involving National Health Service (or equivalent) patients, their organs, tissues or data, and that the necessary arrangements are in place with partner organisations. Where You also accept the responsibilities of a Sponsor (as defined in the Policy Framework), You must also ensure that the requirements for Sponsors are met.

RGC 2.12 Peer review is an integral part of the application process and ensures research of the highest calibre is funded. Investigators and named Researchers on this Grant are expected to make all reasonable efforts to undertake the peer review of proposals for UKRI when invited to do so, unless there is a conflict of interest or the proposal is outside of their area of expertise.

RGC 2.13 By accepting this Grant You are confirming that the Grant Holder has not already received competitively obtained research or support funding from any source, for the same research Project that this Grant has been awarded by Us to support. We reserve the right to terminate the Grant should We find that the Grant Holder has been or is in receipt of the aforementioned duplicate funding, either before or during the Grant Period.

RGC 3 Research Governance
RGC 3.1 Research Ethics, Misconduct and Conflicts of Interest

RGC 3.1.1 You are responsible for ensuring that ethical issues relating to the Project are identified and brought to the attention of the relevant approval or regulatory body. Before any such work requiring approval begins, approval must have been granted by the relevant body.

RGC 3.1.2 You must follow Our Policy and Guidelines on Governance of Good Research Conduct at: https://www.ukri.org/about-us/policies-and-standards/research-integrity/ and ensure that the requirements set out in the Concordat to Support Research Integrity (2012) are met. In particular, You are responsible for ensuring all necessary permissions are obtained before the Project begins, that there is clarity in roles and responsibility among Grant Holders, Research Workers, and Third Parties, as well as

investigating and reporting unacceptable research conduct. Any potential conflicts of interest in research identified at the point of application must be declared to Us and subsequently managed.

RGC 3.2 Use of Animals in Research
You must comply with the provisions of the Animals (Scientific Procedures) Act 1986, and any amendments, where applicable and ensure that all necessary licences are in place before any work requiring approval takes place. You should also follow the guidance set out in "Responsibility in the use of animals in bioscience research": https://www.nc3rs.org.uk/responsibility-use-animals-bioscience-research

RGC 3.3 Health and Safety
You are responsible for ensuring a safe working environment for all individuals associated with the Project, both on and off-site, and for meeting all regulatory and legislative health and safety requirements.

We reserve the right to require You to undertake a safety risk assessment in individual cases where health and safety may be an issue, and to monitor and audit the actual arrangements made. In the event of a serious incident (e.g. death) we require that you inform us for risk purposes.

RGC 3.4 Equality, Diversity and Inclusion
You are expected to ensure that equality, diversity and inclusion is considered and supported at all stages throughout the performance of the Project, in alignment with Our policies and principles at: https://www.ukri.org/about-us/policies-and-standards/equality-diversity-and-inclusion/ for equality, diversity and inclusion. Your approach to supporting equality, diversity and inclusion is expected to exceed all relevant legal obligations, including but not limited those of the Equality Act 2010.

RGC 3.5 Safeguarding
All relevant safeguarding legislation must be adhered to, We particularly draw your attention to child protection legislation and the Modern Slavery Act 2015. You must have sufficient policies and/or processes in place in order to foster Safeguarding.

RGC 3.6 Bullying and Harassment
You must have clear, well-publicised policies, processes and training in place consistent with good practice as recommended by the Advisory, Conciliation and Arbitration Service's (ACAS) 'Bullying and Harassment in the Workplace: A Guide for Managers and Employers'.

RGC 3.7 Whistleblowing
You must have clear, well-publicised policies and processes in place consistent with good practice recommended by the National Audit Office Assessment Criteria for Whistleblowing policies.

RGC 4 Use of Grant
RGC 4.1 We reserve the right to vary the value of the Grant during its lifetime in accordance with the GDP Deflators published by HM Government or to take into account any other Government decisions affecting the funding available to UKRI.

RGC 4.2 With the exception of RGC 4.3, Directly Incurred and Exceptions funds must not be used to meet the costs of an activity that will fall outside the Grant Period.

RGC 4.3 Expenditure may be incurred prior to the start of the Grant and be subsequently charged to the Grant, provided that it does not precede the date of the Offer Letter.

RGC 4.4 Transfers of funds between fund headings are permitted only within and between Directly Incurred and Exceptions costs, excluding equipment, at the rate applicable for the heading as set out in the award letter. Funds may only be transferred into studentship stipend or fees to supplement an existing studentship post on the Grant. You must not transfer funds to create new posts without prior approval from UKRI. Directly Incurred and Exceptions funds must not be used to meet costs on any other Grant or activity.

Funds can only be transferred and used to meet the cost of activity or activities that meet the agreed aims and objectives of the project. While approval does not need to be sought from Us for transfer of funds (excluding the creation of new posts), We reserve the right to query any expenditure outlined in the Final Expenditure Statement which has not been incurred in line with the Standard Terms and Conditions of Grant and any Specific Terms and Conditions of Grant.

RGC 4.5 Costs associated to Students must not be charged to the Grant. These costs must be met by other resources held by You, which can include UKRI Training Grants if the student holds a UKRI studentship. Students are able to undertake paid work within the institution as casual assistance, this should be evidenced with a clear audit trail and should not form part of the formal studentship training.

RGC 5 Starting Procedures
RGC 5.1 You must formally accept the Grant by completing and returning the Offer Acceptance within 10 working days of the issue of the Offer Letter.

RGC 5.2 You must submit the Start Confirmation within 42 (calendar) days of the Project starting. The date entered on the Start Confirmation will be the Official Start Date of the Grant. The Official Start Date may be delayed by up to 3 months from the start date shown in the Offer Letter, but the duration of the Grant will remain unchanged. The Grant may lapse if the Project is not started within 3 months of the start date in the Offer Letter. The start of the Grant may precede the start date shown in the Offer Letter, but must not be earlier than the issue date of the Offer Letter itself.

RGC 6 Extensions
RGC 6.1 The duration of the Grant ("Grant Period") may be extended after the Official Start Date by up to 12 months without

additional funding subject to Our prior written approval. For further information, see the UKRI fEC Grant Guidance document.

RGC 6.2 For Fellowship Grants, the Grant Period may also be extended to cover familial leave, extended jury service or paid sick leave after the Official Start Date for a period in line with the Terms and Conditions of the Fellow's employment. For further information, see the UKRI fEC Grant Guidance document.

RGC 7 Monitoring
RGC 7.1 Changes to Project
You must inform and consult Us if there are any significant changes that may affect the progress, delivery or State Aid status of the Project. No substantive changes to the experimental design of a project involving the use of animals or human participation, which might affect the ethical characteristics of the award, are permitted without the prior approval of UKRI.

If You propose to make significant changes to the Project, UKRI may require revised proposals for its approval and reserves the right to make a new Grant in place of the existing Grant, or to revise, retain or terminate the existing Grant.

RGC 7.2 Transfer of a Grant to another Research Organisation
RGC 7.2.1 The Grant may be transferred to another eligible organisation, providing that it can provide a suitable environment to enable the project to be successfully completed; this will be subject to prior written approval of UKRI. Written agreement to this is required from both the relinquishing and receiving organisations.

RGC 7.2.2 Grant funding will not be revised following transfer. The receiving organisation must confirm that it will provide any additional resources needed to complete the project by returning an Offer Acceptance.

RGC 7.3 Change of Grant Holder
RGC 7.3.1 For Research Grants, You must submit any proposed changes of Grant Holder to UKRI for approval via the Grant Maintenance facility in Je-S.

RGC 7.3.2 For Fellowship Grants, changes to the Grant Holder are not permitted. In the event of the research fellow's resignation or other termination of their employment, the Grant will terminate automatically.

RGC 7.4 Research Monitoring and Evaluation
RGC 7.4.1 You must use Our nominated online system to submit information for monitoring and evaluation purposes on the outputs and outcomes and impacts of the Project during and for some years after the expiry of the Grant Period. Further information on reporting requirements can be found on the UKRI website: https://www.ukri.org/funding/information-for-award-holders/research-outcomes/help-and-guidance/. Failure to comply with the reporting requirements will result in suspension of Grant payments and no further proposals will be considered by UKRI where the Grant Holder is named as the Principal or Co-Investigator.

RGC 7.4.2 Exceptionally We may require a separate End of Award Report on the conduct and outcome of the Project. If required You must submit the report within 3 months of the end of the Grant Period. No further application from a Grant Holder will be considered while an End of Award Report is overdue.

RGC 7.4.3 We reserve the right to call for periodic updates on the Project's progress or to visit the Project team, or request participation in evaluation studies. The Grant Holder must make all reasonable efforts, if so invited, to respond to requests for information or to attend events or activities organised by UKRI concerning the research undertaken, including requests or events after the end of the Grant Period.

RGC 7.5 Disclosure and Inspection
RGC 7.5.1 We shall be entitled to inspect any financial or other records and procedures associated with the Grant as are reasonably required to verify the regularity and propriety of Grant expenditure, or to appoint any other body or individual for the purpose of such inspection. This includes expenditure by Third Parties.

RGC 7.5.2 If We request it, You must provide a statement of account for the Grant, independently examined by an auditor who is a member of a recognised professional body, certifying that the expenditure has been incurred in accordance with the Grant Terms and Conditions.

RGC 7.5.3 You must report to us any investigations and their outcomes into research misconduct associated with the Grant in advance of any enquiry whether informal or formal, and upon request, provide information on Your management of research integrity and ethics as described at: www.ukri.org/about-us/policies-and-standards/research-integrity/. In addition, You must provide details of any retractions or withdrawal of submissions/publications, any allegations, proven or not, of cases of fraud and any other complaint or investigation into dishonesty, fraudulent activities or business misconduct, by any regulatory body or the police into Your activities or those of Your staff.

RGC 7.5.4 We will undertake periodic reviews of Research Organisations within the Funding Assurance Programme to seek assurance that Grants are managed in accordance with the Terms and Conditions under which they are awarded.

RGC 8 Staff
RGC 8.1 Employment
You are wholly responsible for staff funded from the Grant, including Research Fellows, and accept all duties owed to and responsibilities for these staff, including, without limitation, their terms and conditions of employment, and their training and supervision, arising from the employer/employee relationship. You must appoint a Research Fellow as an employee for the full duration of the award.

RGC 8.2 Career Development
You are expected to adopt the principles, standards and good practice for the management of research staff set out in the 2019

Concordat to Support the Career Development of Researchers, including any subsequent amendments.

RGC 8.3 Maternity, Paternity, Adoption and Parental Leave
RGC 8.3.1 At the end of the Grant Period We will reimburse costs incurred by You to cover any additional net parental leave costs that cannot be met within the announced grant cash limit including Statutory Maternity, Paternity and Adoption Pay for staff, within the Directly Incurred and Exceptions fund headings. This will be payable only for the percentage of time that the staff are contracted on the Grant.

RGC 8.3.2 Within the announced grant cash limit, the Grant may be used to meet the costs of making a substitute appointment and/or extending the Grant to cover a period of parental leave for staff within the Directly Incurred and Exceptions fund headings (as outlined above). Directly Allocated and Indirect funds will not be increased as a result of such extensions.

RGC 8.3.3 You will be responsible for any liability for parental leave pay for staff supported by the Grant outside the original Grant Period.

RGC 8.3.4 Fellows are entitled to take parental leave in accordance with the terms and conditions of their employment. We will consider requests for a Fellowship Grant to be placed in abeyance during the absence of the Research Fellow for parental leave, and the period of the Fellowship extended by the period of leave. We will also consider requests to continue the Fellowship on a flexible or part-time basis to allow the Research Fellow to meet caring responsibilities.

RGC 8.4 Sick Leave
RGC 8.4.1 At the end of the Grant Period, We will reimburse You for any additional net sick leave costs that cannot be met within the announced Grant cash limit for staff within the Directly Incurred and Exceptions fund headings, except where You have already recovered these costs by claiming Statutory Sick Pay from HMRC. This will be payable only for the percentage of time that the staff are contracted on the Grant.

RGC 8.4.2 Within the announced grant cash limit, the Grant may be used to meet the costs of making a substitute appointment and/or extending the Grant to cover a period of sick leave for staff within the Directly Incurred and Exceptions fund headings (as outlined RGC 8.4.1). Directly Allocated and Indirect funds will not be increased as a result of such extensions.

RGC 8.4.3 You will be responsible for any liability for sick leave pay for staff supported by the Grant outside the original Grant Period.

RGC 8.4.4 Where there is a continuous period of sick leave in excess of 3 months, You may request approval for a substitute appointment to safeguard progress on the Project. Where a Research Assistant has been on sick leave in excess of 3 months, You must comply with all obligations to consider reasonable adjustments before making a substitute appointment. Where a Research Assistant has been on sick leave for an aggregate (not necessarily continuous) period in excess of 3 months, where this is due to a single condition or a series of related conditions, You may request an extension to the duration of the project.

RGC 8.4.5 Fellowship Grants: Fellows are entitled to take sick leave in accordance with the Research Organisation's terms and conditions. If requested, consideration will be given to allowing a fellowship grant to be placed in abeyance during the absence of the Research Fellow due to sick leave, and the period of the fellowship extended by the period of sick leave. The additional salary costs for the fellow (pro rata to their percentage FTE on the fellowship) should be claimed, as necessary, at the end of the extended period.

RGC 9 Equipment
RGC 9.1 Procurement of Equipment
The procurement of equipment, consumables and services, including maintenance, must comply with all relevant national and EU legislation and consideration must be given to the energy and waste implications of all procurements. For contracts over £25,000, excluding VAT, professionally qualified procurement staff must be consulted before the procurement process begins. Any proposal to purchase equipment in the last 6 months of the Grant must be pre-approved by UKRI.

RGC 9.2 Ownership of Equipment
You must inform us if the need for the equipment diminishes substantially or it is not used for the purpose for which it was funded during the Grant Period. We reserve the right to determine the disposal of such equipment and to claim the proceeds of any sale. Any proposal to transfer ownership of the equipment during the period of the Grant requires the prior approval by UKRI.

RGC 9.3 Equipment Data
All new equipment purchased over £138,000 (£115,000 ex VAT) must be registered on the "Equipment.data" national database.

RGC 10 Financial Reporting
RGC 10.1 You are accountable for funds dispersed and are responsible for the timely and accurate submission of all expenditure reports required under the Terms and Conditions of Grant, including the submission of an expenditure statement within 3 months of the end of the Grant Period. We are entitled to require You to provide supplementary information in support of an interim or final expenditure statement. Once an expenditure statement has been received and the expenditure incurred has been reconciled against payments made, it will be considered as final. Any unspent funds will be recovered.

RGC 10.2 You must retain all accounting information relating to the Grant for the current financial year plus the subsequent six years after the submission date of the final expenditure statement.

RGC 10.3 If We send an Annual Statement to return showing payments made by UKRI during the previous financial year for all the Grants You hold, You must complete and return the statement by the specified deadline.

RGC 11 Sanctions

RGC 11.1 We reserve the right to impose financial sanctions and/or additional measures if You do not comply with Your obligations as set out in these Standard Terms and Conditions of Grant and any Specific Terms and Conditions of Grant.

RGC 11.2 If the End of Award Report (if required) or the Financial Expenditure Statement is not received within 3 months of the end of the Grant Period, UKRI will recover 20% of expenditure incurred on the Grant. All payments will be recovered if the report or statement is not received within 6 months of the end of the Grant. You may appeal against a sanction, but must do so within 60 days of the pay run in which the sanction was imposed.

RGC 11.3 Where an Organisation is deemed to be non-compliant in the application of TRAC, a minimum sanction of 75% of the non-compliant rate may be applied, where an Organisation is applying rates which are materially inaccurate (>10% variance on any single rate). These sanctions would only apply to future applications, until a time that UKRI Funding Assurance are satisfied that remedial measures are implemented.

RGC 12 Exploitation and Impact
RGC 12.1 Unless otherwise agreed, all intellectual property shall belong to the party that generates them. Where the Grant is associated with more than one Research Organisation and/or other project partners, the basis of collaboration between the organisations including ownership of intellectual property and rights to exploitation, is expected to be set out in a formal collaboration agreement.

RGC 12.2 You are responsible for ensuring that all parties engaged in the research make every reasonable effort to ensure that the intellectual assets obtained in the course of the research, whether protected by intellectual property rights or not, are used to the benefit of society and the economy.

RGC 12.3 In individual cases, We reserve the right to retain ownership of intellectual assets, including intellectual property (or assign it to a third party under an exploitation agreement) and to arrange for it to be exploited for the national benefit and that of the Research Organisation involved.

RGC 12.4 The Grant Holder shall, subject to the procedures laid down by the Research Organisation, publish the results of the research funded by the Grant in accordance with normal academic practice and Our policy on Open Access: https://www.ukri.org/files/legacy/documents/rcukopenaccesspolicy-pdf/. Other forms of media communication, including media appearances, press releases and conferences, must acknowledge the support received from Us, quoting the Grant reference number if appropriate.

RGC 13 Disclaimer
RGC 13.1 UK Research and Innovation accepts no liability, financial or otherwise, for expenditure or liability arising from the research funded by the Grant except as set out in these Terms and Conditions, or otherwise agreed in writing.

RGC 13.2 UKRI reserves the right to amend the payment profile at its discretion. You will be advised, in advance, of any such change. Changes to payment profiles may affect the overall value of the Grant.

RGC 13.3 UKRI reserves the right to terminate the Grant at any time, subject to reasonable notice and to any payment that We agree may be necessary to cover outstanding and unavoidable commitments. If a Grant is terminated or reduced in value, no liability for payment, redundancy or any other compensatory payment for the dismissal of staff funded by the Grant will be accepted, but, subject to the provisions of RGC 10 Financial Reporting, negotiations will be held with regard to other contractual commitments and concerning the disposal of assets acquired under the research grant.

RGC 13.4 Where studies are carried out in an NHS Trust or equivalent, the Trust or equivalent has a duty of care to its patients. UK Research and Innovation does not accept liability for any failure in the Trust's duty of care, or any negligence on the part of its employees.

RGC 14 Status
RGC 14.1 The Terms and Conditions of Grant which include these Standard Terms and Conditions of Grant and the Specific Terms and Conditions of Grant will be governed by the laws of England and Wales and all matters relating to the Terms and Conditions will be subject to the exclusive jurisdiction of the courts of England and Wales.

RGC 14.2 If any provision of these Terms and Conditions is found by a court or other legitimate body to be illegal, invalid or unreasonable, it will not affect the remaining Terms and Conditions which will continue in force.

RGC 14.3 The Terms and Conditions of Grant contain the whole agreement between UKRI and the Research Organisation in relation to the Grant and neither party intends that any of these Terms and Conditions should be enforceable by any third party.

Annex A
Definitions

Co-Investigator: A person who assists the Grant Holder in the management and leadership of the Project.

Council: Any of the bodies listed under the Introduction.

Directly Allocated Costs: Costs of resources used by the Project that are shared by other activities. They are charged on the basis of estimates rather than actual costs and do not represent actual costs on a project by project basis.

Directly Incurred Costs: Costs that are explicitly identifiable as arising from the conduct of the Project which are charged as the cash value actually spent and are supported by an audit record.

End of Award Report: A report which the Grant Holder must provide at the end of the Grant Period, detailing the outputs, outcomes and impacts of the project to date.

Exceptions: Directly Incurred Costs that Councils fund at 100% of fEC subject to actual expenditure incurred, or items that are outside fEC.

Fellowship Grant: An award made through a fellowship competition providing a contribution to the support of a named individual. It covers the cost of the time dedicated by the fellow to their personal research programme, and may or may not include research support costs.

Full Economic Costs (fEC): A cost which, if recovered across an organisation's full programme, would recover the total cost (direct, indirect and total overhead) including an adequate recurring investment in the organisation's infrastructure.

Funding Assurance Programme: A programme of visits and office based tests by UKRI to seek assurance that grant funds are used for the purpose for which they are given and that grants are managed in accordance with the terms and conditions under which they are awarded Gran Support for a proportion of the full economic costs of the Project. A Grant may be either a Research Grant or a Fellowship.

Grant Holder: The person to whom the Grant is assigned and who has responsibility for the intellectual leadership of the Project and for the overall management of the research funded by the Grant. The Grant Holder is either the Principal Investigator (in the case of a Research Grant) or a Research Fellow (in the case of a Fellowship Grant).

Grant Period: The duration of time between the Project start and end date.

Grant Terms and Conditions: The Standard Terms and Conditions of Grant together with the Specific Terms and Conditions of Grant that together comprise the basis on which the Grant is awarded to the Research Organisation.

Indirect Costs: Non-specific costs charged across all projects based on estimates that are not otherwise included as Directly Allocated Costs. They include the costs of the Research Organisation's administration such as personnel, finance, IT, legal, general laboratory, office consumables, library and some departmental services.

Je-S: Joint Electronic Submissions system used for the submission of Grant related information.

Offer Acceptance: A document to be completed and returned by the Research Organisation either accepting or declining the Grant.

Grant Offer Letter / Offer Letter: An official document setting out specific details of the Grant, including the Project start and end date, Grant value and any Specific Terms and Conditions of the Grant as required by the relevant Council.

Official Start Date: The official start date of the Grant, as set out in the Start Confirmation.

Project: The project funded by the Grant as set out in the Offer Letter.

Research Grant: A contribution to the costs of the research Project which has been assessed as eligible for funding through the procedures established by the relevant Council.

Research Organisation (RO)/Grant Awardee: The organisation to which the Grant is awarded and which takes responsibility for the management of the Project and accountability for funds provided.

Research Worker: Any person or third party working in any capacity on the Project.

Specific Terms and Conditions of Grant/Specific Conditions: The specific conditions of grant required in addition to the Standard Terms and Conditions on a Grant by an individual Council of UKRI.

Standard Conditions of Grant/Standard Conditions: The Standard Terms and Conditions of Grant published on UKRI's website at: www.ukri.org/funding/information-for-award-holders/grant-terms-and-conditions/

Start Confirmation: Confirmation of the date on which the Project commences, as notified by the Research Organisation to UKRI.

Studentship: The term used for the funding award made by a Research Organisation to a student for the purpose of undertaking postgraduate training leading to the award of a postgraduate degree.

Third Party: Any person/organisation to which the award holding RO passes on any of the Grant funds awarded by the Council.

Transparent Approach to Costing (TRAC): An agreed methodology used by universities and other higher education bodies for calculating full economic costs.

Annex B

Information Sources

These Grant Terms and Conditions should be read in conjunction with the following sources. In the event of any conflict the terms of these Conditions should prevail:

1) UKRI Use of grant proposal information addendum: www.ukri.org/files/funding/tcs/grants-addendum-pdf/
2) UKRI Privacy Notice:www.ukri.org/privacy-notice/
3) UKRI Grant Terms and Conditions web page: www.ukri.org/funding/information-for-award-holders/grant-terms-and-conditions/
4) State Aid: Including but not limited to Articles 107 to 109 of the Treaty on the Functioning of the European Union, the General Block Exemption Regulation and any Enabling Regulation, as amended from time to time
5) De Minimis Aid: Commission Regulation (EU) No 1407/2013
6) General Block Exemption Regulation: Commission Regulation EU No. 651/2014
7) Department for Business Innovation and Skills: The State Aid Manual
8) UKRI International Due Diligence Guidance: www.ukri.org/files/funding/due-diligence-guidance-for-ukros-pdf/
9) Concordat for Engaging the Public with Research: www.ukri.org/public-engagement/research-council-partners-and-public-engagement-with-research/embedding-public-engagement/
10) UK Policy Framework for Health and Social Care Research
11) Policy and Guidelines on Governance of Good Research Conduct: www.ukri.org/about-us/policies-and-standards/research-integrity/
12) Concordat to Support Research Integrity (2012)
13) Animals (Scientific Procedures) Act 1986
14) Responsibility in the use of animals in bioscience research guidance: https://www.nc3rs.org.uk/responsibility-use-animals-bioscience-research
15) UKRI Policies and Principles for Equality, Diversity and Inclusion: www.ukri.org/about-us/policies-and-standards/equality-diversity-and-inclusion/
16) Equality Act 2010
17) Modern Slavery Act 2015
18) Advisory, Conciliation and Arbitration Service (ACAS) 'Bullying and Harassment in the Workplace: A Guide for Managers and Employers'
19) UKRI FEC Grant Guidance: https://www.ukri.org/funding/information-for-award-holders/grant-terms-and-conditions/
20) Research Outcome Reporting Requirements: www.ukri.org/funding/information-for-award-holders/research-outcomes1/help-and-guidance/
21) Research Integrity: www.ukri.org/about-us/policies-and-standards/research-integrity/
22) 2019 Concordat to Support the Career Development of Researchers
23) Open Access Policy: www.ukri.org/files/legacy/documents/rcukopenaccesspolicy-pdf/

MRC Additional Terms and Conditions

The MRC additional terms and conditions of funding supplement those of UKRI. These conditions set out operational, legislative and ethical requirements relating to medical research. The MRC reserves the right to vary these additional terms and conditions.

Research organisations and award holders* have absolute responsibility for ensuring all required licenses, approvals, permissions and consent are in place before any research is undertaken and that these are followed.

*Award Holders are all MRC Grant Holders and recipients of MRC Unit and Institute funding (programme leaders).

MRC reserves the right to audit at any time without prior notice:

-That required licenses, approvals, permissions and consent are in place, or were in place when the activity occurred.

-Compliance with the terms and conditions set out here

AC1 Responsibilities of the Research Organisation: Clinicians

The research organisation is responsible for ensuring all clinicians
supported by MRC funding are aware they are individually responsible for maintaining appropriate professional indemnity insurance. This should be with a professional defence organisation for any activities not covered by NHS indemnity arrangements or by additional provision made by the research organisation. MRC will not meet the costs of such cover.

The research organisation is responsible for ensuring any honorary clinical contracts required by clinical staff have been obtained prior to the start of the research.

The MRC expects the research organisations to abide by the 'UK clinical academic training in medicine and dentistry: principles and obligations' (https://mrc.ukri.org/news/browse/improving-support-for-clinical-academics/).

AC2 Clinical Responsibilities

Clinical award holders (Clinical Research Training Fellowships, Clinician Scientist Awards, Senior Clinical Fellowships or Clinical Academic Research Partnerships) may not work more than the time commitment for clinical duties stated in their proposal. For the majority, this will equate to up to 20% (on average over the lifetime of the grant) of their normal working hours, which they may choose to spend on NHS clinical sessions, teaching and demonstrating, or research activities beyond the scope of their fellowship. Exceptions are made for surgeons and fellows undertaking patient-oriented research, who may undertake up to 40% of their time on these duties. This is not in addition to the six hours per week all research staff supported full-time by an MRC grant or fellowship may undertake under RGC 8 of the UKRI Terms and Conditions of Research Council fEC Grants (https://www.ukri.org/funding/information-for-award-holders/grant-terms-and-conditions/).

AC3 Mouse Strains

MRC supports a central repository of mouse strains - the MRC mouse Frozen Embryo and Sperm Archive (FESA) at Harwell.

Award holders are expected to contact FESA to highlight mouse strains engineered, or characterised using MRC funds, and are encouraged to deposit these strains with the archive.

Depositors retain ownership of strains and there is currently no charge for depositing strains to make them freely available to the academic community.

FESA aims to ensure that valuable mouse strains are safeguarded, that the need to maintain colonies of live mice for long periods of time is reduced, and that the significant investment in engineering strains is capitalised upon fully. MRC award holders planning mouse research should contact FESA at the earliest opportunity.

For help with the requirements of AC5-AC13 please contact MRC Regulatory Support Centre: https://mrc.ukri.org/research/facilities-and-resources-for-researchers/regulatory-support-centre/.

AC4 Human Participants in Research

MRC expects all research involving human participants to be undertaken in accordance with its policies and guidance available from https://mrc.ukri.org/research/policies-and-guidance-for-researchers/. These include:

-Good Research Practice (2012);

Using information about people in health research (2016-17)

-Human Tissue and Biological Samples for Use in Research (2014);
-Medical research involving adults who cannot consent (2007);
-Medical Research Involving Children (2004);
-Guidelines for the management of global health trials (2017).

Research organisations and award holders have absolute responsibility for ensuring that investigations being undertaken within NHS premises, nursing or residential homes or NHS service establishments, schools, or any other organisations, do not take place without the explicit approval of the appropriate authority in advance.

AC5 Approvals

Independent Research Ethics Committee approval is required for research that involves human participants (whether patients or healthy volunteers) or records. In the case of research involving NHS patients, premises or records, this will be an NHS Research Ethics Committee (REC). Such approval is also required for certain studies of human tissues. Further guidance on when NHS REC approval is required can be found at (http://www.hra-decisiontools.org.uk/ethics/).

In the case of social science research, the MRC recommends that award holders follow the ESRC Framework for Research Ethics (revised 2015, https://esrc.ukri.org/funding/guidance-for-applicants/research-ethics/ which highlights the responsibility of the research organisation for ensuring that the research is subject to appropriate ethics review. In some case's this review is required by an NHS REC, for further guidance please see (https://www.hra.nhs.uk/approvals-amendments/what-approvals-do-i-need/research-ethics-committee-review/).

MRC does not need to be routinely notified by the award holder of amendments required by a regulator or a REC unless they relate to urgent safety measures and/or substantially change the research approved for funding by the MRC.

AC6 Payments and incentives in research

Payments to healthy volunteers participating in clinical research are allowable, provided that the payment is for expense, time and inconvenience and is not at a level which would induce people to take part in studies against their better judgement. Further guidance on payments and incentives in research can be found at https://www.hra.nhs.uk/about-us/committees-and-services/nreap/.

AC7 Clinical Trials

When research involves MRC-funded clinical trials, award holders must act in accordance with MRC policy on UK clinical trials regulations (https://mrc.ukri.org/research/policies-and-guidance-for-researchers/clinical-research-governance/clinical-trials-regulations/), in relation to ethical, sponsorship, reporting, monitoring and publication requirements. Research involving trial oversight and management for MRC-funded clinical trials conducted in lower and middle income countries (LMICs), should refer to the MRC guidelines for management of global health trials https://mrc.ukri.org/funding/science-areas/global-health-and-international-partnerships/funding-partnerships/joint-global-health-trials/.

-An independent Trial Steering Committee, and in most cases a Data Monitoring Committee, must be set up to oversee the conduct of the trial, with an MRC representative acting as an observer. In exceptional circumstances for particularly low risk trials, a researcher may seek approval from the MRC Programme Manager for more limited TSC and/or DMC oversight structures.

-MRC-funded trials must be registered with an International Standardised Randomised Control Trial Number (ISRCTN) on the ISRCTN Registry (http://www.isrctn.com/). The unique identification number must be used in publications and provided to MRC by adding it to Researchfish within a year of the trial starting. Failure to provide this number will result in suspension of funding.

-Results of MRC-funded trials (whether positive or negative) must be published without unreasonable delay and within 24 months of completion of the study. Results should be reported in accordance with the recommendations in the CONSORT statement (http://www.consort-statement.org/). Before results are published, they must be discussed by the Trial Steering Committee.

-Any contribution to an MRC-funded trial by another body, such as a pharmaceutical company (donation of drugs etc.), must be the subject of a collaboration agreement between the parties (see AC20).

AC8 UK Policy Framework for Health and Social Care Research

Research involving NHS (or HSC in Northern Ireland) patients, their organs, tissues or data which falls within the scope of the UK Policy Framework for Health and Social Care Research (https://www.hra.nhs.uk/planning-and-improving-research/policies-standards-legislation/uk-policy-framework-health-social-care-research/) must comply with MRC policy on the health departments' research governance framework (https://mrc.ukri.org/research/policies-and-guidance-for-researchers/clinical-research-governance/health-departments-research-governance/).
MRC requires research organisations to ensure sponsorship responsibilities are clearly identified, the research undertaken complies with the requirements of the employing organisation set out in the UK policy framework, and that agreements and systems are in place with NHS Trusts and other partner organisations, including commercial organisations, to comply with the framework. Systematic documentation of key decisions and approvals, particularly in relation to work with patients, their organs, tissues and data is crucial.

AC09 Medical Records

When research involves the use of medical records, the award holder must act in accordance with the principles set out in data protection legislation (https://ico.org.uk/for-organisations/guide-to-data-protection/introduction-to-data-protection/some-basic-concepts/) and the NHS requirements to protect patient confidentiality. Advice on these requirements is available from the MRC Regulatory Support Centre.

All researchers handling personal data must have clearly established obligations to maintain confidentiality (e.g. formalised within policy written by their research organisations or through professional codes of conduct).

Research involving identifiable patient-level data will require NHS Research Ethics Committee approval and may also require additional approvals. In England and Wales research involving identifiable patient-level data, without patient consent, is covered by "Section 251" of The National Health Service Act 2006 and requires additional approval via the Health Research Authority's Confidentiality Advisory Group (https://www.hra.nhs.uk/approvals-amendments/what-approvals-do-i-need/confidentiality-advisory-group/). In Scotland, decisions on disclosure of identifiable patient-level data are made by Caldicott Guardians (see https://www.informationgovernance.scot.nhs.uk/pbpphsc/ for further details).

AC10 Data Sharing

Award holders must comply with the MRC policy on research data sharing (https://mrc.ukri.org/research/policies-and-guidance-for-researchers/data-sharing/) along with the MRC policy on sharing of research data from population and patient studies (https://mrc.ukri.org/research/initiatives/health-and-biomedical-informatics/access-governance-and-ethics/).

When research involves clinical trials, clinical intervention studies, public health intervention studies or observational studies award holders must comply with the MRC policy on open research data from clinical trials and public health interventions (https://mrc.ukri.org/research/policies-and-guidance-for-researchers/open-research-data-clinical-trials-and-public-health-interventions/).

AC11 Removal, Use or Storage of Human Tissue

Award holders whose research involves the removal, use or storage of human tissue as specified in the relevant legislation must:

-comply with the appropriate legislation, ie the Human Tissue Act 2004 and/or the Human Tissue (Scotland) Act 2006;

-follow the relevant standards and Codes of Practice issued by the Human Tissue Authority (HTA) (the MRC Regulatory Support Centre https://mrc.ukri.org/research/facilities-and-resources-for-researchers/regulatory-support-centre/ has summarised these);

-follow the MRC guidance detailed within the Policies and Guidance for Researchers (https://mrc.ukri.org/research/policies-and-guidance-for-researchers/) to download the Human Tissue and Biological Samples for Use in medical Research PDF.

Where research involves the use of human tissues and cells to treat patients (human application), award holders must also:

-comply with the Human Tissue (Quality and Safety for Human Application) Regulations 2007;

-work within the applicable regulations and standards as dictated by the Human Tissue Authority, Medicines and Healthcare products Regulatory Agency (MHRA), Human Fertilisation and Embryology Authority and Health Research Authority. The UK Stem Cell Tool Kit (www.sc-toolkit.ac.uk/home.cfm), gives guidance on applicable regulatory routes, and the MHRA Innovation Office (www.gov.uk/government/groups/mhra-innovation-office), provides a regulatory advice service for regenerative medicine.

When research involves the use of human fetal tissue, or non-fetal products of conception (ie amniotic fluids, umbilical cord, placenta or membranes), researchers should follow the guidance set out in Consent Code of Practice issued by the HTA (in particular, please see paragraphs 141-143 at https://www.hta.gov.uk/hta-codes-practice-and-standards-0).

When research involves procedures for the removal of human tissue at post-mortem examination, researchers must also follow guidance issued by the Health Departments and Local Health Authorities.

AC12 Stem Cells

Award holders whose research involves human stem cell lines (both embryonic and adult) must:

-Abide by the UK Code of Practice for the use of Human Stem Cell lines (Code of Practice can be downloaded from the MRC website: https://mrc.ukri.org/research/policies-and-guidance-for-researchers/uk-stem-cell-bank-steering-committee/).
-Ensure that they hold all relevant licenses, accreditations and approvals from, and abide by the Codes of Practice issued by, but not limited to, the Human Fertilisation and Embryology Authority (HFEA; see AC10), the Human Tissue Authority (HTA; see AC12), the Health Research Authority (HRA; for research ethics, gene therapy and confidentiality; see AC6, AC7, AC8), the Medicines and Healthcare products Regulatory Agency (MHRA; see AC6, AC7, AC8), the EU Tissue and Cells Directive (where applicable).

In the case of research involving human embryonic stem cells:

-Deposit a sample of every human embryonic stem cell line derived with MRC funding in the UK Stem Cell Bank; applications to deposit or access banked stem cell lines must be approved by the Steering Committee for the UK Stem Cell Bank and for the Use of Stem Cell Lines (https://mrc.ukri.org/research/policies-and-guidance-for-researchers/uk-stem-cell-bank-steering-committee/).

-Not pass samples of human embryonic stem cell lines to third parties other than those approved by the Steering Committee for the UK Stem Cell Bank and for the Use of Stem Cell Lines and/or the HFEA.

-Not take human embryonic stem cell lines out of the UK unless approved by the Steering Committee for the UK Stem Cell Bank and for the Use of Stem Cell Lines and/or the HFEA.

-Scientists from overseas wishing to conduct human embryonic stem cell research in the UK as visiting workers must provide a written statement from their home institution, outlining that as the employer of the visiting worker they take on the responsibilities of ensuring their employee works to and complies with the requirements of the UK Governance landscape, set out in the UK Code of Practice.

-Send copies of publications to the UK Stem Cell Bank, and agree that the UK Stem Cell Bank may post summaries of published results on their web site.

-Assist the MRC and the UK Stem Cell Bank, on request, with public engagement activities.

AC13 Human Fertilisation

When research involves the use of human gametes, embryos or human admixed embryos researchers must act in accordance with the Human Fertilisation and Embryology Act 1990 as amended in 2008 and 2015 (the Human Fertilisation and Embryology (Mitochondrial Donation) Regulations). This includes obtaining a research licence to undertake activities covered by the Act. Further information can be obtained from https://www.hfea.gov.uk/.

AC14 Ionising radiation

Under the Ionising Radiation (Medical Exposure) Regulations 2017 and the Ionising Radiation (Medical Exposure) (Northern Ireland) Regulations 2018, Research Ethics Committee approval is required where participants are to be exposed to ionising radiation as part of their involvement in medical or biomedical, diagnostic or therapeutic, research.

Research studies involving the administration of radioactive substances must also be approved by the Administration of Radioactive Substances Advisory Committee (ARSAC).

For further guidance: https://www.hra.nhs.uk/planning-and-improving-research/policies-standards-legislation/ionising-radiation/.

AC15 Genetic Modification

In accordance with the Genetically Modified Organisms (Contained Use) Regulations 2014, research organisations and individuals undertaking genetic modification must be registered with the Health and Safety Executive (HSE), undertake risk assessment and seek consent where appropriate.

Researchers who carry out genetic modification should be familiar with the legislative requirements and with the Scientific Advisory Committee on Genetic Modification (Contained Use) guidance. Advice can be obtained from HSE Head Office or from your nearest HSE Office and Knowledge Centre (https://www.hse.gov.uk/contact/).

AC16 Dangerous Pathogens

Research organisations accommodating projects involving the use of dangerous pathogens must comply with the safeguards recommended by the Advisory Committee on Dangerous Pathogens (https://www.hse.gov.uk/aboutus/meetings/committees/acdp/) in their guidance 'Infection at work: controlling the risk', 'Biological Agents: the principles, design and operation of containment in a level 4 facility' (https://www.hse.gov.uk/biosafety/information/).

AC17 Controlled Drugs and Substances

When research requires the use of one or more of the drugs controlled under the Psychoactive Substances Act 2016 or the Misuse of Drugs Act, 1971 and its subsequent amendments, researchers must hold an appropriate Home Office licence in accordance with the most up to date Regulations.

AC18 Open Access Policy - Publication Repositories

To comply with the UKRI Policy on Open Access (see RGC 12.4 of the UKRI Research Council fEC Terms and Conditions) the MRC requires all publications to be deposited at the earliest opportunity, and certainly within six months of publication, in Europe

PubMed Central (europepmc.org/). This applies both during and after the period of funding. The condition is subject to compliance with publishers' copyright and licensing policies. Whenever possible, the article deposited should be the published version. For more information see (https://mrc.ukri.org/research/policies-and-guidance-for-researchers/open-access-policy/).

AC19 MRC Industry Collaboration Agreement

It is a condition of MRC Industry Collaboration Agreement (MICA) awards that the PI/research organisation must provide MRC Head Office with a copy of the collaboration agreement, signed by all partners, within 3 months of the date of this letter and prior to the award start date. The agreement must be consistent with the Heads of Terms submitted with the application. The grant cannot be activated, and payments, made until this document has been submitted and approved by the MRC.
