# Supplementary material for: A randomised controlled trial to compare clinical and cost-effectiveness of an online parent-led treatment for child anxiety problems with usual care in the context of COVID-19 delivered in Child and Adolescent Mental Health Services in the UK (Co-CAT): a study protocol for a randomised controlled trial
Source: Trials. 2022 Nov 16;23:942. doi: 10.1186/s13063-022-06833-5 (PMC9667839; doi:10.1186/s13063-022-06833-5)
Supplement: Supplementary file 3 — Additional file 3. NIHR204435 Intend to Fund letter. Original funder approval (NIHR). [file 13063_2022_6833_MOESM3_ESM.pdf]

NIHR | National Institute for Health and Care Research

**CENTRAL COMMISSIONING FACILITY**
Grange House
15 Church Street
Twickenham
TW1 3NL

Tel: 020 8843 8000
Fax: 020 8843 8001
Email: ccf@nihr.ac.uk
www.nihr.ac.uk/ccf

Friday 27 May 2022

To whom it may concern,

I am writing to confirm that the National Institute for Health Research (NIHR) is funding the following research project at the University of Warwick subject to final contract signature:

**NIHR204435: Enabling Child and Adolescent Mental Health Services (CAMHS) to provide efficient remote treatment for child anxiety problems in the COVID-19 context**

The lead investigators are Professor Cathy Cresswell.

The project has a start date of **01 July 2022** and a budget of **£233,410.49**

Yours sincerely,

Fiona Giles
Programme Manager
