# Supplementary material for: A randomised controlled trial to compare clinical and cost-effectiveness of an online parent-led treatment for child anxiety problems with usual care in the context of COVID-19 delivered in Child and Adolescent Mental Health Services in the UK (Co-CAT): a study protocol for a randomised controlled trial
Source: Trials. 2022 Nov 16;23:942. doi: 10.1186/s13063-022-06833-5 (PMC9667839; doi:10.1186/s13063-022-06833-5)
Supplement: Supplementary file 4 — Additional file 4. Original ethics approval. [file 13063_2022_6833_MOESM4_ESM.pdf]

Ymchwil Iechyd a Gofal Cymru
Health and Care Research Wales

NHS
Health Research Authority

Professor Cathy Creswell
Professor of Developmental Clinical Psychology (Full-time); NIHR Research Professor; Hon Consultant Clinical Psychologist
University of Oxford, Department of Experimental Psychology
Radcliffe Observatory Quarter
Oxford
OX2 6GG

Email: approvals@hra.nhs.uk
HCRW.approvals@wales.nhs.uk

04 September 2020

Dear Professor Creswell

HRA and Health and Care Research Wales (HCRW) Approval Letter

**Study title:**	**Child Anxiety Treatment in the context of COVID-19 (Co-CAT):Enabling Child and Adolescent Mental Health Services (CAMHS) to provide efficient remote treatment for child anxiety problems**
**IRAS project ID:**	**288074**
**Protocol number:**	**1.0**
**REC reference:**	**20/HRA/4431**
**Sponsor**	**University of Oxford / Clinical Trials and Research Governance**

I am pleased to confirm that **HRA and Health and Care Research Wales (HCRW) Approval** has been given for the above referenced study, on the basis described in the application form, protocol, supporting documentation and any clarifications received. You should not expect to receive anything further relating to this application.

Please now work with participating NHS organisations to confirm capacity and capability, in line with the instructions provided in the "Information to support study set up" section towards the end of this letter.

How should I work with participating NHS/HSC organisations in Northern Ireland and Scotland?
HRA and HCRW Approval does not apply to NHS/HSC organisations within Northern Ireland and Scotland.

If you indicated in your IRAS form that you do have participating organisations in either of these devolved administrations, the final document set and the study wide governance report (including this letter) have been sent to the coordinating centre of each participating nation. The relevant national coordinating function/s will contact you as appropriate.

Please see IRAS Help for information on working with NHS/HSC organisations in Northern Ireland and Scotland.

How should I work with participating non-NHS organisations?
HRA and HCRW Approval does not apply to non-NHS organisations. You should work with your non-NHS organisations to obtain local agreement in accordance with their procedures.

What are my notification responsibilities during the study?

The standard conditions document "*After Ethical Review – guidance for sponsors and investigators*", issued with your REC favourable opinion, gives detailed guidance on reporting expectations for studies, including:

- Registration of research
- Notifying amendments
- Notifying the end of the study

The HRA website also provides guidance on these topics, and is updated in the light of changes in reporting expectations or procedures.

Who should I contact for further information?
Please do not hesitate to contact me for assistance with this application. My contact details are below.

Your IRAS project ID is **288074**. Please quote this on all correspondence.

Yours sincerely,

Kevin Ahmed (On behalf of Rebecca Evans)
Approvals Manager

Email: approvals@hra.nhs.uk

Copy to: *CTRG*

List of Documents

The final document set assessed and approved by HRA and HCRW Approval is listed below.

Document	Version	Date
Contract/Study Agreement template [Site Agreement]		28 August 2020
Covering letter on headed paper [Co-CAT IRAS cover letter]		27 August 2020
Evidence of Sponsor insurance or indemnity (non NHS Sponsors only) [Client Information Letter]		24 July 2020
Interview schedules or topic guides for participants [Topic guide - Therapist]	Version 1.0	21 August 2020
Interview schedules or topic guides for participants [Topic Guide - Parents]	Version 1.0	21 August 2020
IRAS Application Form [IRAS_Form_27082020]		27 August 2020
Letter from funder [MRC Funding Confirmation]	Version 1.0	21 August 2020
Letter from sponsor [C Creswell signed Sponsor Letter]		27 August 2020
Non-validated questionnaire [AE Form - Child]	Version 1.0	21 August 2020
Non-validated questionnaire [AE Form - Parent]	Version 1.0	21 August 2020
Non-validated questionnaire [Demographic Information - Parent]	Version 1.0	21 August 2020
Non-validated questionnaire [Demographic Information - Pareny]	Version 1.0	21 August 2020
Non-validated questionnaire [Therapist Demographic Information]	Version 1.0	26 August 2020
Non-validated questionnaire [Therapist Treatment Logs]	Version 1.0	26 August 2020
Non-validated questionnaire [Therapist Supervision Logs]	Version 1.0	26 August 2020
Non-validated questionnaire [Therapist Experience of Treatment]	Version 1.0	26 August 2020
Organisation Information Document [Organisation Information Document - Word Format]	Version 1.0	27 August 2020
Other [OSI Overview and Screenshots]	Version 1.0	26 August 2020
Other [Gantt Chart]	Version 1.0	21 August 2020
Other [Privacy Notice - Parents]	Version 1.0	26 August 2020
Other [Privacy Notice - Therapists]	Version 1.0	26 August 2020
Other [Letter to GP]	Version 1.0	26 August 2020
Participant consent form [Parent Consent Form - self]	Version 1.0	26 August 2020
Participant consent form [Parent Consent Form - child]	Version 1.0	26 August 2020
Participant consent form [Child Assent Form]	Version 1.0	26 August 2020
Participant consent form [Therapist Consent Form]	Version 1.0	26 August 2020
Participant information sheet (PIS) [Parent Information Sheet]	Version 1.0	26 August 2020
Participant information sheet (PIS) [Child Information Sheet]	Version 1.0	26 August 2020
Participant information sheet (PIS) [Therapist Information Sheet]	Version 1.0	26 August 2020
Referee's report or other scientific critique report [MRC Reviewers Feedback]	Version 1.0	21 August 2020
Research protocol or project proposal [Co-CAT Research Protocol]	Version 1.0	26 August 2020
Sample diary card/patient card [Parent Health Usage Diary]	Version 1.0	26 August 2020
Schedule of Events or SoECAT [SoECAT - Excel format]	Version 1.0	21 August 2020
Summary CV for Chief Investigator (CI) [Chief Investigator CV]	Version 1.0	21 August 2020
Summary, synopsis or diagram (flowchart) of protocol in non technical language [Protocol Appendix 2]	Version 1.0	26 August 2020
Validated questionnaire [CAIS-P]		
Validated questionnaire [CAIS-C]	Version 1.0	26 August 2020

Validated questionnaire [CHU-9D-P]		
Validated questionnaire [CEIS (baseline)]	Version 1.0	26 August 2020
Validated questionnaire [Credibility and Expectation of Improvement Scale (adapted)]	Version 1.0	26 August 2020
Validated questionnaire [CSRI (baseline)]	Version 1.0	26 August 2020
Validated questionnaire [CSRI (14 weeks)]	Version 1.0	26 August 2020
Validated questionnaire [CSRI (26 weeks)]	Version 1.0	26 August 2020
Validated questionnaire [EQ5D-P]		
Validated questionnaire [GBO]		
Validated questionnaire [ORS]		
Validated questionnaire [PAS]		
Validated questionnaire [RCADS-C]		
Validated questionnaire [RCADS-P]		
Validated questionnaire [SDQ]		
Validated questionnaire [SRS]		

IRAS project ID	288074

Information to support study set up

The below provides all parties with information to support the arranging and confirming of capacity and capability with participating NHS organisations in England and Wales. This is intended to be an accurate reflection of the study at the time of issue of this letter.

Types of participating NHS organisation	Expectations related to confirmation of capacity and capability	Agreement to be used	Funding arrangements	Oversight expectations	HR Good Practice Resource Pack expectations
All sites will perform the same research activities therefore there is only one site type.	Research activities should not commence at participating NHS organisations in England or Wales prior to their formal confirmation of capacity and capability to deliver the study.	An Organisation Information Document has been submitted and the sponsor is intending to use a model non-commercial agreement with sites.	No study funding will be provided to sites as per the Organisational Information Document	The Sponsor has confirmed that a Principal Investigator is required at site.	No Honorary Research Contracts, Letters of Access or pre-engagement checks are expected for local staff employed by the participating NHS organisations. Where arrangements are not already in place, research staff not employed by the NHS host organisation undertaking any of the research activities listed in the research application would be expected to obtain a Letter of Access based on standard DBS checks and occupational health clearance.

Other information to aid study set-up and delivery

This details any other information that may be helpful to sponsors and participating NHS organisations in England and Wales in study set-up.
The applicant has indicated they intend to apply for inclusion on the NIHR CRN Portfolio.
